# A universal AC electrokinetics-based strategy toward surface antifouling of underwater optics

**DOI:** 10.1038/s41598-024-66251-2

**Published:** 2024-07-12

**Authors:** Hao Jiang, Yan Wang, Fei Du, Stefan Stolte, Uwe Specht, Georg R. Pesch, Michael Baune

**Affiliations:** 1 College of Chemistry and Chemical Engineering, Qingdao University, Qingdao, 266071 People’s Republic of China; 2 Institute of Water Chemistry, Dresden University of Technology, 01069 Dresden, Germany; 3 The Fraunhofer Institute for Manufacturing Technology and Advanced Materials, 28359 Bremen, Germany; 4 School of Chemical and Bioprocess Engineering, University College Dublin, Belfield, Dublin 4, Ireland; 5 Center for Environmental Research and Sustainable Technology, University of Bremen, 28359 Bremen, Germany

**Keywords:** Surface antifouling, Underwater optics, Alternative current electrokinetics, Asymmetric electrode array, Numerical simulations, Environmental sciences, Chemistry, Engineering, Materials science

## Abstract

The practical applications of underwater optical devices, such as cameras or sensors, often suffer from widespread surface biofouling. Current antifouling techniques are primarily hindered by low efficiency, poor compatibility, as well as environmental pollution issues. This paper presents a transparent electrode coating as antifouling system of underwater optics as potential substitute for alternating current electrokinetic (ACEK)-based systems. A strong-coupling model is established to predict the Joule heating induced fluid flows and the negative dielectrophoretic (nDEP) effect for mobilizing organisms or deposited sediments on optic surfaces. The performance of the proposed antifouling system is numerically evaluated through simulations of electrostatic, fluid and temperature fields as well as trajectories of submicron particles, which is then experimentally verified and found to be in good agreement. A parametric study revealed that the degree of electrodes asymmetry is the key factor affecting the flow pattern and therefore the overall performance of the system. This ACEK-based universal strategy is expected to shed light on designing high performance and non-toxic platforms toward energy-efficient surface antifouling applications of underwater optics.

## Introduction

Surface biofouling is the main hindrance for long-term, autonomous monitoring applications of underwater optics^1^. Biofouling, induced by the undesired settlement and accumulation of microorganisms, plants, and animals on submerged transparent surfaces such as sensor components, viewing windows, lighting and camera systems, causes for instance data drift of sensor monitoring, decrease in transmittance of light measurement, blurred video and images acquired by cameras and even irreparable damage to optical instruments^2^. The biofouling could be suppressed and eradicated using biocides^3^, mechanical cleaning systems^4^, UV irradiation^5^, or non-stick coating^6^. However, all these methods suffer from different problems in the application on underwater optics.

Employing a biocide system can protect surfaces from fouling, however, it requires additional cleaning steps and causes pollution to the environment inducing adverse impact on biological monitoring^7^. For example, an antifouling system proposed by Pinto et al.^8^ can effectively suppress biofouling by generating sufficient chlorine using electrolysis of seawater. However, the generated chlorine might cause adverse effects to other species in the environment. In addition, the produced bubbles could disturb the function of optical instrument. Mechanical methods adopted in some marine instruments, including water scrapers or brushes, have advantages of high effectiveness, low cost and easy maintenance. However, these techniques suffer from compatibility problems when working with circular optical, lighting or other sensitive sensor systems^9^. MacKenzie et al.^10^ successfully hindered formation of biofilm and growth of most organisms using periodic ultraviolet irradiation. However, this approach requires additional lighting structures, which may affect the working efficiency of optical devices. Surface Acoustic Wave (SAW) devices, characterized by high efficiency and sensitivity, have been demonstrated promising applications in underwater antifouling. However, their effectiveness in deep-sea environments can be profoundly impacted by environmental variables such as pressure fluctuations, temperature changes, marine biological activities, and mechanical noise. Therefore, a transparent, non-toxic approach that can be applied to underwater optical surfaces without affecting the analytical performance of the instrument is desired.

The term alternating-current electrokinetics (ACEK) summarizes techniques to induce fluid flow, such as AC electrothermal (ACET) and AC electroosmosis (ACEO), as well as to manipulate particles, e.g., dielectrophoresis (DEP), in inhomogeneous electric fields. ACEK has been widely applied in liquid pumping and mixing^11^, solid and liquid separation^12^, and solids classification^13^ due to its high selectivity and controllability. The fluid flow induced by ACEK depends on various interrelated factors, such as input voltage, field frequency, electrical conductivity, dielectric constant, viscosity, and more importantly, the scale of the system^14^. At low electric frequency (e.g. ~ 10^1^–10^5^ Hz) or electric conductivity (e.g. ~ 10^–4^–10^–3^ S/m), the net migrating charges at the solid–liquid interface move under the action of the inhomogeneous electric field and pull the fluid flow, resulting in ACEO. When the electric frequency is higher than 100 kHz or the medium conductivity is larger than 0.002 S/m, fluid motion induced by ACET force instead dominate the behavior due to the highly compressed electric double layer (EDL)^15^. Joule heating in inhomogeneous electric fields generates local temperature gradients in the system, which in turn trigger fluid flow, mainly ACET flow and buoyancy flow. The induced fluid flow can disturb the manipulation of particles in microfluidic devices, therefore in early studies, low voltage or cooling plates were used to reduce the Joule heating interferences^16^. In recent years, the ACET flow has been applied in microfluidic devices for fluid mixing and pumping. For example, Kunti et al.^17^ investigated a micromixer driven by a high efficiency ACET micropump, which utilizes the asymmetry of the electrode array and the electric field. Through optimization of the parameter configuration, a mixing efficiency of 97.25% was attained, accompanied by uniform and homogeneous mixing quality. Zhang et al.^18^ proposed a two-phase ACET micropump utilizing a coplanar asymmetric electrode array. The generated asymmetric vortices and unidirectional fluid flow induced by ACET in the vicinity of the electrode surface significantly enhanced the fluid rate. By optimizing design parameters, the proposed two-phase ACET structure can achieve up to 50% faster fluid flow than the corresponding single-phase structure.

Dielectrophoresis is mainly used as a technology for separation and manipulation of micron and submicron particles. It has been widely applied in analytical and biomedical fields because of its unique advantages of being label free and controllable and because it shows high sensitivity and selectivity^19^. Recent studies have shown that DEP can also be adopted in membrane filtration and wastewater treatment processes to inhibit membrane surface pollution. Du et al.^20^ used a pair of bare grid electrodes as the membrane support and insulating electrodes arranged on the opposing side to intensify a crossflow membrane filtration process, which achieved an extension of the membrane working time by 3.3 times. Ren et al.^21^ comprehensively considered the effects of insulator-based dielectrophoretic (iDEP) and ACET on the antifouling performance of nanoporous membranes (NPMs). They found that iDEP offers a good repulsive effect on nanoparticles within the nanopores, while ACET vortex flow had an inhibitory effect on the antifouling performance. This inhibitory effect induced by symmetrical ACET flow was attributed to the symmetry of the electric field strength, which inhibits the repulsion of the nanoparticles from the nanopores. To generate net flow by ACET flow, an asymmetric electrode array is required. While many researches are focused on actuating fluid/droplets^18,22^ as mentioned above, ACET can synergize with DEP to repel and remove micron-/nanoparticles from the surface of target objects. To the best of the authors’ knowledge, the application of asymmetric electrode arrays in ACEK-based surface antifouling has not been reported.

Here, we present a novel underwater surface antifouling system which is based on a combination of dielectrophoresis and AC electrothermal fluid flow. We chose indium tin oxide (ITO) as electrode material as it is transparent and therefore ideally suited to be applied in optical devices. An asymmetric electrode array is used to generate DEP forces on the particles as well as to induce fluid flow by AC electrothermal movement. The combination of ACET and DEP will repel and wash away organisms and sediments from underwater surfaces. A theoretical model is established to predict the Joule heating induced fluid flow as well as the DEP effect for levitating pollutant particles from optical surfaces. Submicron polystyrene (PS) particles, which are physically similar to those marine organisms/cells^23^, are taken as model pollutants to evaluate the performance of the proposed antifouling system. We systematically study the fluid flow induced by ACET and buoyancy and further explore the design and operating parameters that affect the antifouling performance. We use a comprehensive approach through both experiments and simulation to characterize our novel anti-fouling system. This lab-based study serves to spotlight the possibilities of our approach for fouling mitigation in submerged surfaces, especially in saline (maritime) environments. This is, because the ACET effect increases with increasing conductivity, making this approach ideally suitable for high-conductive media.

## Numerical simulation and experimental validation

### Theory

#### Forces on the fluid

##### ACET

The temperature gradient induced by Joule heating in an inhomogeneous electric field generates local variations in conductivity and permittivity. The interaction between the inhomogeneity of conductivity and permittivity and electric field induces the fluid flow. For aqueous systems with high conductivity (such as seawater), a strong driving force can be generated to induce ACET vortices under a small input voltage. The volumetric ACET force can be expressed as^14^

1 $$ {\mathbf{F}}_{{{\text{ET}}}} = \frac{1}{2}\frac{{\varepsilon_{{\text{m}}} \left( {\alpha - \beta } \right)}}{{1 + \left( {\omega \tau } \right)^{2} }}\left( {\nabla T \cdot {\mathbf{E}}} \right){\mathbf{E}} - \frac{1}{4}\varepsilon_{m} \alpha \left| {\mathbf{E}} \right|^{2} \nabla T, $$

where ${\mathbf{E}} = - \nabla \varphi$ is the electric field vector (effective) and $\varphi$ is the electric potential, $\omega = 2\pi f$ is the angular frequency of the AC electric field, ${\text{f}}$ is the frequency of the electric field, $\tau = \varepsilon_{{\text{m}}} /\sigma_{{\text{m}}}$ is the charge relaxation time, $\varepsilon_{{\text{m}}} = \varepsilon_{{\text{r, m}}} \varepsilon_{0}$ and $\sigma_{{\text{m}}}$ are the permittivity and conductivity of the medium, respectively, $\varepsilon_{{\text{r, m}}}$ is the relative permittivity of the medium, $\varepsilon_{0} = 8.854 \times 10^{ - 12} {\text{F/m}}$ is the permittivity of vacuum, ${\text{T}}$ is temperature of the system, and $\alpha$ and $\beta$ are the two thermal diffusion coefficients. For aqueous solutions, the numerical values of $\alpha$ and $\beta$ can be approximated as $\alpha = (\partial \varepsilon_{{\text{m}}} /\partial T)/\varepsilon_{{\text{m}}} \approx$ − 0.04 (K^−1^) and $\beta = (\partial \sigma_{{\text{m}}} /\partial T)/\sigma_{{\text{m}}} \approx$ 0.02 (K^−1^)^24^.

##### Buoyancy

In addition to ACET, Joule heating (which occurs in highly conductive aqueous solutions) can cause local density differences in the fluid and further trigger buoyancy-driven natural convection. In an ACEK system with characteristic dimensions (i.e., electrode width or spacing) greater than 1 mm, the buoyancy body force drives flow recirculation, which surpasses ACET flow and dominates the overall fluid motion^25^. The expression of buoyancy force per unit volume is

2 $$ {\mathbf{F}}_{{\text{b}}} = \Delta \rho_{{\text{m}}} {\mathbf{g}} = \left( {\partial \rho_{{\text{m}}} /\partial T} \right)\Delta T{\mathbf{g}}, $$

where ${\uprho }_{{\text{m}}}$ is the density of the medium, and ${\text{g}}$ is the gravitational acceleration^26^.

#### Forces on particles

##### DEP

When suspended particles are subjected to a non-uniform electric field, their induced dipole moment interacts with the electric field, leading to DEP motion. The direction of the particle motion is determined by the difference of the polarizability between particle and suspension medium. If particles are more polarizable than the suspension medium, they will experience positive DEP forces (pDEP) and move towards the region of high electric field intensity. Conversely, particles which are less polarizable than the suspension medium will experience negative DEP forces (nDEP) and thus are repelled by the high electric field intensity region. In conductive aqueous suspension, many solid particles show nDEP due to their lower polarizability. The time-averaged DEP force acting on a spherical particle can be calculated by^15^

3 $$ {\mathbf{F}}_{{{\text{DEP}}}} = 2\pi a^{3} \varepsilon_{{\text{m}}} {\text{Re}} \left[ {K\left( \omega \right)} \right]\nabla \left| {\mathbf{E}} \right|^{2} , $$

where *a* is the radius of the spherical particle, ${\text{K}}\left( {\upomega } \right)$ is the frequency-dependent Clausius–Mossotti factor^27^

4 $$ K\left( \omega \right) = \frac{{\varepsilon_{{\text{p}}}^{*} - \varepsilon_{{\text{m}}}^{*} }}{{\varepsilon_{{\text{p}}}^{*} + 2\varepsilon_{{\text{m}}}^{*} }}. $$

Here $\varepsilon_{{\text{p}}}^{*}$ and $\varepsilon_{{\text{m}}}^{*}$ are the complex permittivity of the particle and medium, respectively, $\varepsilon^{*} = \varepsilon - j\sigma /\omega$ with the permittivity $\varepsilon = \varepsilon_{{\text{r}}} \varepsilon_{0}$, the relative permittivity $\varepsilon_{{\text{r}}}$ and the electrical conductivity $\sigma$.

##### Drag

When relative motion exists between fluid and solid, the fluid exerts a drag force on the surface of the solid. For spherical particles, the drag force can be expressed as follows^28^:

5 $$ {\mathbf{F}}_{{{\text{Drag}}}} = \frac{1}{2}\pi a^{2} \rho_{{\text{m}}} C_{{\text{D}}} \left( {{\mathbf{u}} - {\mathbf{v}}} \right)^{2} , $$

where *C*_D_ is the particle Reynolds number-dependent drag coefficient, **u** is the fluid velocity, and **v** is the particle velocity.

##### Gravity

In a gravitational field, a solid is subject to the buoyant forces exerted by surrounding fluid. For spherical particles, this buoyant force is

6 $$ {\mathbf{F}}_{{\text{g}}} = \frac{4}{3}\pi a^{3} \left( {\rho_{{\text{p}}} - \rho_{{\text{m}}} } \right){\mathbf{g}}, $$

where $\rho_{{\text{p}}}$ is the density of the particle.

### Design principles

**Figure 1:**
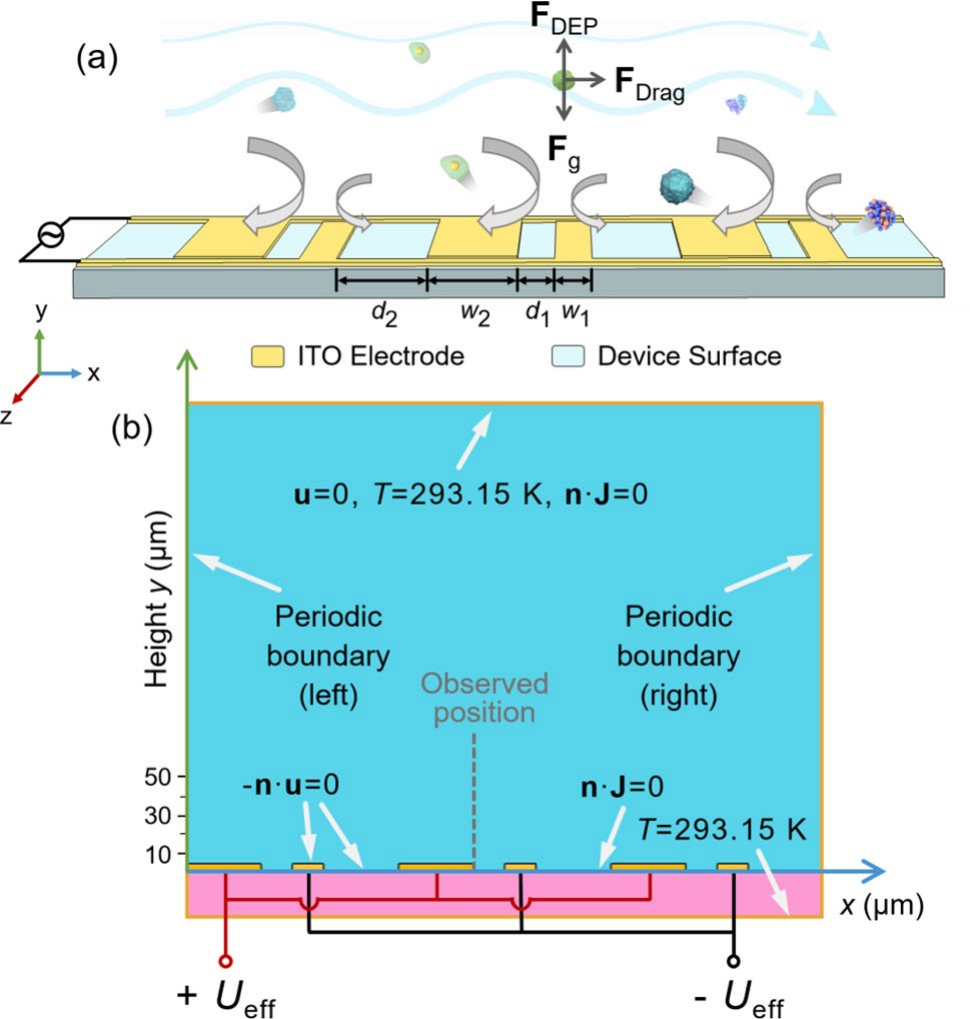
(**a**) Sketch of the proposed underwater surface antifouling system with (**b**) computational domain and boundary conditions for the numerical simulations.

The design principle of the proposed underwater surface antifouling system is illustrated in Fig. 1. Note that this proposed technology is intended to be used for the practical cleaning of underwater surfaces. In this work, we only explore the feasibility and dependencies in a lab-based setup as a proof-of-concept study. Specifically, an array of asymmetric interdigitated electrode is designed. Transparent ITO is chosen as electrodes on the surface of underwater devices to generate DEP force and fluid flows. Many particles have lower polarizability compared to seawater and therefore subject to nDEP, such particles will be moved away from the surface. Meanwhile, the Joule heating induced flows, i.e., ACET and buoyancy, will generate asymmetric vortices in the vicinity of the electrode surfaces and unidirectional fluid flow away from electrodes. As a result, through the synergistic interaction between the asymmetrical vortex flow and the nDEP effect, pollutant particles deposited on the surface of optic devices are lifted upwards and drifted out of the system by the unidirectional fluid flow. To better illustrate the ability of the surface antifouling strategy, we evaluate the antifouling performance of the proposed system by a combined effect of the net fluid flow velocity on surfaces and the nDEP velocity of pollutant particles.

### Numerical simulation

#### Governing equations

The finite element software package COMSOL Multiphysics 5.5 was utilized to conduct numerical simulations and assess the efficacy of an ACEK-based surface antifouling system (see Fig. 1). To simplify the system, we reduce variables by letting the distance between electrodes (*d*_1_) equal to the size of small electrode (*w*_1_), and the distance between electrode pairs (*d*_2_) equal to the size of large electrode (*w*_2_). The aspect ratio *r* is introduced to characterize the degree of asymmetry of the electrode array, given as:

7 $$ r = w_{2} /w_{1} . $$

The simulation framework consists of a two-dimensional model that incorporates electrostatics, heat transfer, and laminar flow. Additionally, the particle tracking module is used to investigate the efficiency of the system on particle trajectories. In a system with strong temperature variations, the flow dynamics of the vortex generated by the electrothermal force deviates significantly from those predicted by conventional models using a small temperature approximation. In light of this, Loire et al.^29^ modified the Poisson equation to solve the electric field and arrived at a convection–diffusion style equation:

8 $$ \nabla^{2} \varphi = \gamma \cdot \nabla \varphi , $$

where the factor $\gamma = - \beta \nabla T$ if $\omega \tau \ll 1$ and $\gamma = - \alpha \nabla T$ if $\omega \tau \gg 1$.

The energy balance equation solves the Joule heating effect under AC electric field by coupling the electric field and the thermal field^30^:

9 $$ \nabla \cdot \left( {k_{{\text{m}}} \left( T \right)\nabla T} \right) + \frac{{\sigma_{{\text{m}}} \left( T \right)}}{2}\left| {\mathbf{E}} \right|^{2} = 0, $$

where $k_{{\text{m}}}$ is the thermal conductivity of the medium related to temperature.

The solution of the flow field is based on the continuity equation and the Navier–Stokes equation^31^:

10 $$ \rho_{{\text{m}}} \left( T \right){\mathbf{u}} \cdot \nabla {\mathbf{u}} = - \nabla P + \nabla \cdot \left( {\eta \left( T \right)\nabla {\mathbf{u}}} \right) + {\mathbf{F}}_{{{\text{ET}}}} + {\mathbf{F}}_{{\text{b}}} , $$

11 $$ \nabla \cdot \left( {\rho_{{\text{m}}} \left( T \right){\mathbf{u}}} \right) = 0, $$

where $\eta$ is the dynamic viscosity of the medium, which is related to the fluid temperature; and *P* is the pressure of the flow field.

Trajectories of particles affected by drag, gravity, and DEP forces are solved by Newton's second law:

12 $$ \frac{{{\text{d}}\left( {m_{{\text{p}}} v} \right)}}{{{\text{d}}t}} = {\mathbf{F}}_{{{\text{Drag}}}} + {\mathbf{F}}_{{\text{g}}} + {\mathbf{F}}_{{{\text{DEP}}}} , $$

where *m*_p_ is the particle mass, and *t* is the time.

#### Computational domain and boundary conditions

In this study, the calculated system can be simplified to a two-dimensional geometry since the electrodes are long compared to their height and width (Fig. 1). The computational domain is composed of three groups of asymmetric plate electrodes embedded on a 1.1 mm thick glass substrate, with periodic boundary conditions applied to both the left and right boundaries. For the electric field, the device surface (excluding the electrode) and the top fluid boundary are electrically insulating with ${\text{n}} \cdot {\text{J}}$ = 0, where ${\text{n}}$ is the unit normal vector and ${\text{J}} = \sigma_{{\text{m}}} {\mathbf{E}}$ is the current density. For the flow field, the fluid velocity at the top boundary is assumed to be stationary with a velocity of **u** = 0, while the fluid velocity on both surface of electrodes and glass substrate are given as slip boundary with $- {\text{n}} \cdot {\text{u}}$ = 0 due to the smoothness of material surfaces. Additionally, since the device is in an infinitely large natural fluid environment (in comparison to the size of the device itself), the upper boundary of the fluid and the lower boundary of the device are considered to be at room temperature (*T* = 293.15 K).

#### Model and mesh independence study

The model used in this study was validated by comparing numerical simulations of the field distributions with literature values by Williams^32^. As presented in Fig. S1, the simulated electric field and flow patterns are in good agreement with those in the literature, indicating good accuracy of our model. In addition, the fitted relationship between velocity and applied voltage (see Fig. S2) indicates that the flow rate is proportional to the power of 5.28 of the voltage, which conforms to the enhanced model proposed by Loire et al.^29^.

A mesh-independence study was conducted for the model system (see Fig. S3) to obtain reliable simulation results independent of mesh size. The maximum relative deviations of the flow field and electric field between simulation results using different mesh numbers of 426,938 and 329,865 are 1.22% and 1.03%, respectively. Further increasing the mesh number gives a negligible effect on the accuracy of the calculations while significantly increasing computational costs. Therefore, a total mesh number in the computational domain was selected as 329,865 in this study.

### Experimental method

#### Electrodes fabrication

The substrate material consists of 1.1 mm thick glass slides (25 × 75 × 1.1 mm^3^) which are coated with a 650 nm thick ITO layer (BIOTAIN CRYSTAL CO., LIMITED, CHN), as shown in Fig. S4. The surface resistivity is 2 ± 0.5 Ohm/sq. The surface electrodes were created by selective laser ablation. A 100 W Nd:YAG laser (FA Clean-Lasersysteme GmbH) with wavelength 1064 nm was used to scan the samples at a focal length of 160 mm in a 2D scanning system. The power of the laser was set to 5 W, resulting in a circular ITO ablation of 30 µm at the laser focus.

To obtain interdigitated electrodes on the ITO glass slides, a CAD model was created and transferred to the laser. The electrodes were rounded in a semicircle at their ends to avoid electrical voltage peaks. The ITO ablation finally results in the interdigital spaces between the electrodes. With a laser focus distance along the scan direction and between adjacent laser scan lines of 2 µm, sharp-edged electrodes are obtained in the Keyence VHX-600 light microscope shown in Fig. S4. In this way, areas of 1 × 1 cm^2^ (Fig. S4) on the glass slides were generated with electrodes and contact areas being cut out with the laser. The resistance between the electrodes at the contact points was approx. 1.5 ± 0.5 kΩ. The smaller electrode distance *w*_1_ was chosen as 40 µm and *w*_2_ = 200 µm achieve sufficient electrical resistances (> 100 Ω) between adjacent electrodes. The resulting electrode array is shown in Fig. S4.

#### Materials and methods

Fluoresbrite® yellow green polystyrene (PS) particles (Polysciences, Germany) with diameter of 0.75 µm (1.08 × 10^11^ particles/mL, excitation max. = 441 nm, emission max. = 486 nm) were used in experiments. Sodium chloride (NaCl) (Merck Germany) was mixed with water at a certain mass concentration for synthesizing salty water with a defined conductivity, which is measured and determined using a portable conductivity meter (WTW Cond 3110) at room temperature. The concentration of particles in NaCl suspension was 8.64 $\times$ 10^9^ particles/L. 0.005 vol% of Tween 20 (Merck Germany) was added to reduce the interactions between particles and homogenize the suspension. The identical suspension was used in all experiments in this work.

**Figure 2:**
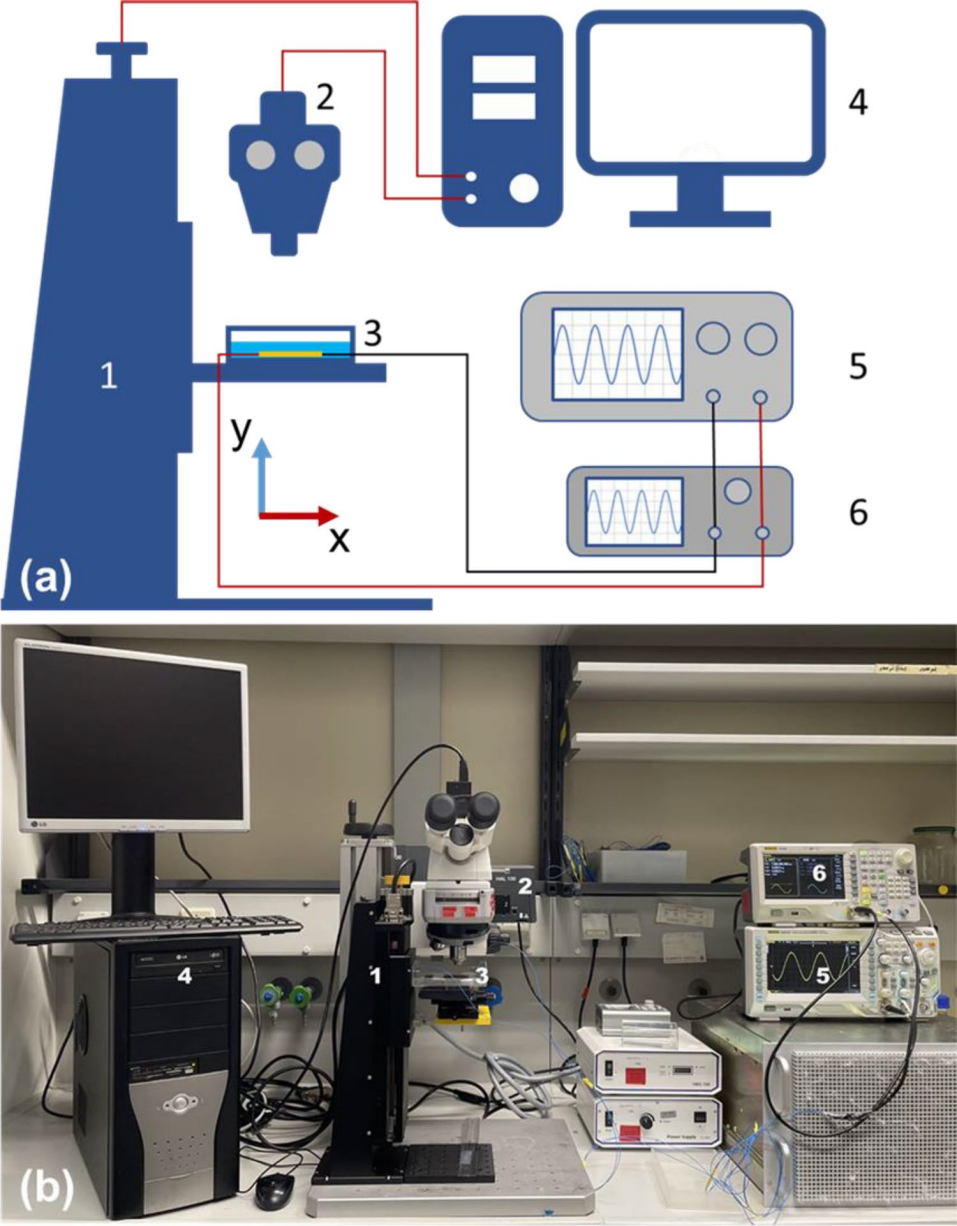
Schematic diagram (**a**) and picture (**b**) of experimental setup, with linear stage (1), fluorescence microscope (2), electrokinetic cell (3), computer (4), oscilloscope (5), and function generator (6).

A high precision linear stage (LIMES150, OWIS) allows precisely moving in the y direction to defined height by the installed motor and controlled by software OWISoft v2.90 (OWIS, Germany). It was used to hold, control, and adjust the height of electrokinetic cell (Fig. S4), which was fixed on the bottom of a sample container (85 $\times$ 130 $\times$ 20 mm^3^) (3 in Fig. 2a). Suspension was added into the sample container with its height maintained at 10 mm from the bottom of sample container. The sample container was mounted on two translation stages (THORLABS, DTS50/M) for adjusting positions in *x* and *z* directions (Fig. 2b). A fluorescence microscope (ZEISS Scope A1) installed with camera (FLIR Grasshopper 3, GS3-U3-51S5C-C), objective lens (EC Epiplan 10×/0.25 M27) and fluoresce filter set (GFP) was used to observe, acquire and save the videos of particles’ motion trajectories to a computer. Function generator (RIGOL DG4062) is applied to provide electrokinetic cell electrical signal with maximal peak-to-peak voltage of 20 V and frequency up to 40 MHz. The signal output from the function generator is monitored using an oscilloscope (RIGOL DS2072A).

After filling particle suspension, the height of zero (*y* = 0) is first found and fixed by focusing the objective of fluorescence microscope on the surface of ITO electrodes. The zero-height position of the setup is defined for the linear stage in its controlling software OWISoft v2.90. By adjusting the relative height of the linear stage through OWISoft and the motor, we can record particle trajectories at different heights from the ITO array. The function generator is switched on to generate electric field at a defined frequency, 1 kHz in this work. The oscilloscope measures the output signal and then record the effective voltage (*U*_eff_) and frequency. The video clips of particles motion are recorded and saved using software FlyCapture 2. The velocity of particles is measured and evaluated using software Tracker, a free video modelling tool built on the opensource physics (OSP) Java framework (https://physlets.org/tracker/).

## Results and discussion

### Simulations of physical field distribution and particle trajectory

**Figure 3:**
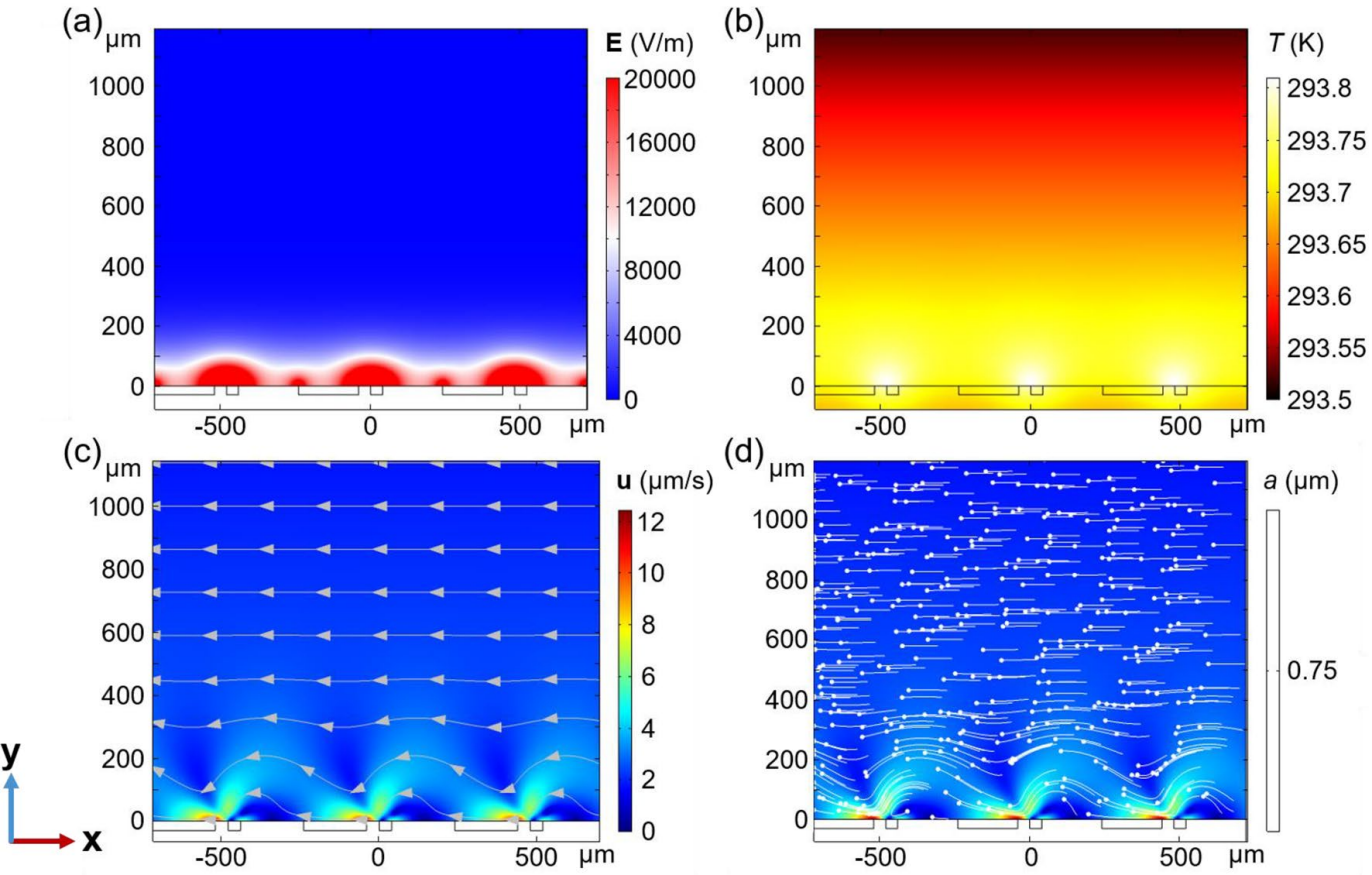
Numerical simulations of distributions of (**a**) electric field, (**b**) temperature, (**c**) velocity, and (**d**) the trajectories of particles (PS, particle size *a* = 0.75 µm) (*w*_1_ = 40 µm, *w*_2_ = 200 µm, *U*_eff_ = 5.66 V, $\sigma_{{\text{m}}}$ = 0.01 S/m, and *f* = 1 kHz).

In this work, a novel underwater surface antifouling system based on asymmetric electrode array is established. The asymmetrical electric field intensity induced by the periodically arranged asymmetric electrode array induces flow on the optical surface. The maximum values of electric field intensity and temperature are observed at the edge of the short electrode *w*_1_, respectively, adjacent to the long electrode *w*_2_ (Fig. 3a,b). Taking a closer look at one pair of electrodes, the fluid flow directs from short electrode (*w*_1_) to large electrode (*w*_2_) (Fig. 3c). Due to the uneven distribution of electric field intensity and temperature gradient, the fluid on the surface of the electrode exhibits an asymmetric flow state, induced by the combined effects of ACET and buoyancy. Despite of the asymmetric flow, the fluid direction does not change (from right to left). The fluid flow asymmetry reduces significantly with increasing height from electrodes surface, in the meantime, a unidirectional flow dominates the overall fluid motion (Fig. 3c). Such the fluid pattern, in conjunction with the nDEP effect experienced by most pollutant particles (here, as an example, 0.75 µm PS), could effectively reduce sedimentation and adhesion of particles to optical surface, and hence suppress fouling on the surface of optical devices. From the particle trajectories (Fig. 3d) and snapshots of particle positions (Fig. S5), it is evident that PS particles are visibly pushed up and removed from the surface of the optical device, primarily due to the nDEP effect (the calculated ${\text{Re}} \left[ {K\left( \omega \right)} \right]$ is shown in Fig. S6) in combination with the unidirectional ACET flow. Besides, the statistical particle residue rate quantitatively demonstrates the antifouling capability of the system (Fig. S7).

To further demonstrate the ability of the asymmetric electrode design on the proposed antifouling system, we compare electric field intensity and flow rate profiles, as well as the velocity at different heights for an asymmetric electrode array and a symmetric electrode array in Fig. S8. Comparatively uniform electric field distributions of the symmetric electrode array give rise to much weaker asymmetric flow in the vicinity of the electrode surfaces as well as negligible fluid flow away from the electrodes (Fig. S8).

### Experimental validation

**Figure 4:**
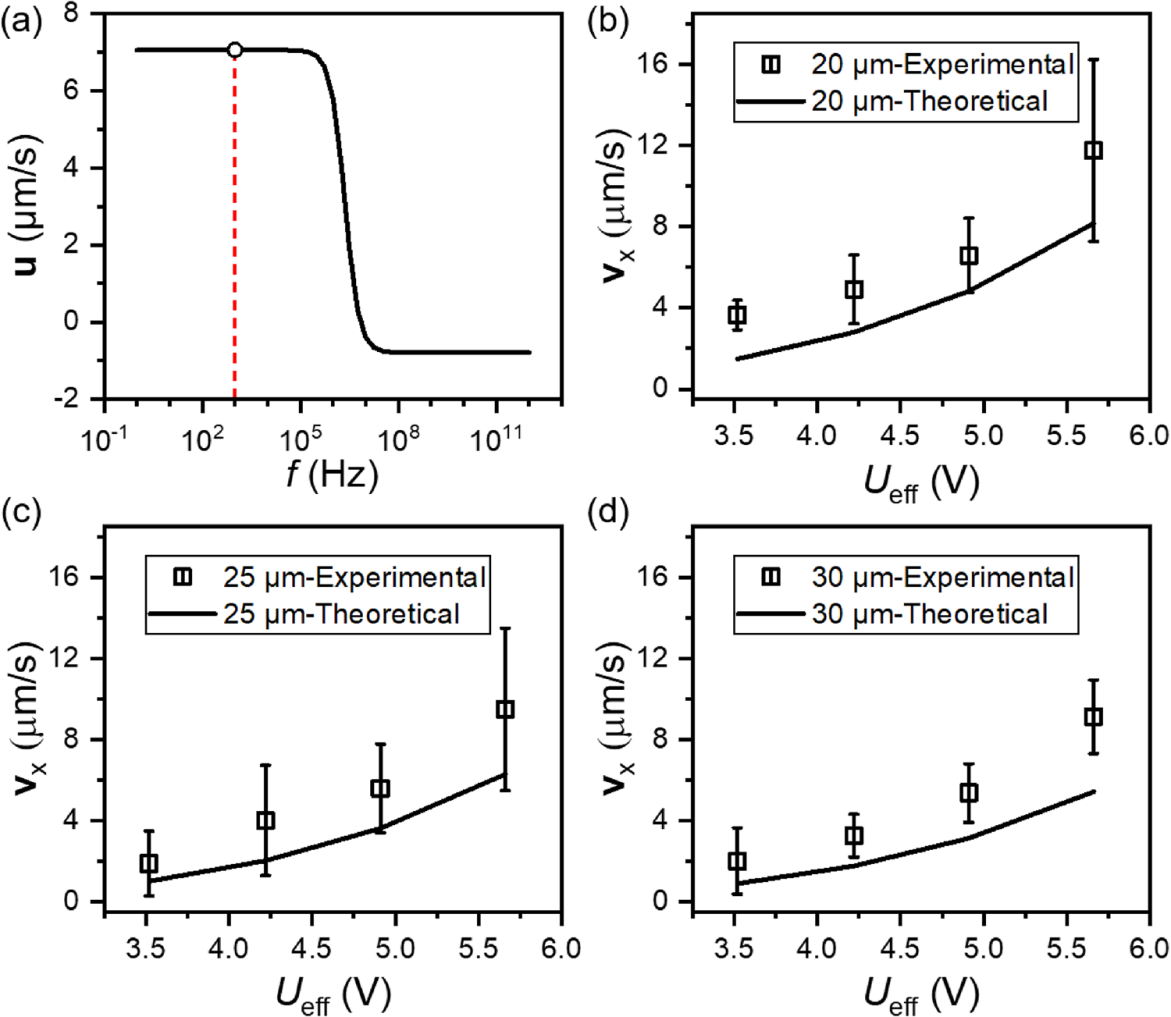
(**a**) Simulated flow rate at 20 µm height with input voltage of *U*_eff_ = 5.66 V as a function for frequency *f*. (**b–d**) Experimentally measured and simulated velocities at different observed heights of 20, 25 and 30 µm above electrodes (as indicated on Fig. 1) (*w*_1_ = 40 µm, *w*_2_ = 200 µm, $\sigma_{{\text{m}}}$ = 0.01 S/m and *f* = 1 kHz).

To verify the feasibility of the antifouling system and the reliability of the numerical simulation results, experimental verification and data measurement of the antifouling system were conducted by observing particle trajectories and measuring their velocity in aqueous medium with the conductivity of 0.01 S/m. As presented in Eqs. (1) and (3), the induced ACET fluid flow depends on the frequency. This was also numerically calculated in Fig. 4a. The frequency used in both simulation and experiments, *f* = 1 kHz, is shown in the graph as red dashed line. It is far lower than the crossover frequency seen in Fig. 4a that causes force transformation and reduction in the fluid flow. Therefore, the frequency dependence of the fluid flow is neglected in this study. Moreover, the effect of ACEO was not considered in the numerical model, because the applied frequency in this work is much higher than the characteristic frequency of ACEO (estimated as 2.87 Hz at 0.01 S/m, which is the threshold to generate ACEO effect).

It can be seen from Fig. 4b–d that the overall velocities increase with voltage input for both numerical simulations and experimental measurements. Experimentally measured velocities of the 0.75 µm PS particles agree well with simulations at different measurement positions. The slight difference between experimental velocities and simulations could be attributed to the measurement error of specific positioning during the experiment and the definition of the baseline for the height (*y* = 0). Further, using periodic boundaries on both left and right sides as well as the assumption of ideal heat dissipation conditions at the bottom of the device during numerical simulations are not consistent with the real experimental situation. These differences in boundary conditions could also explain the small discrepancy between numerical simulations and the obtained experimental data.

### Parametric study

**Figure 5:**
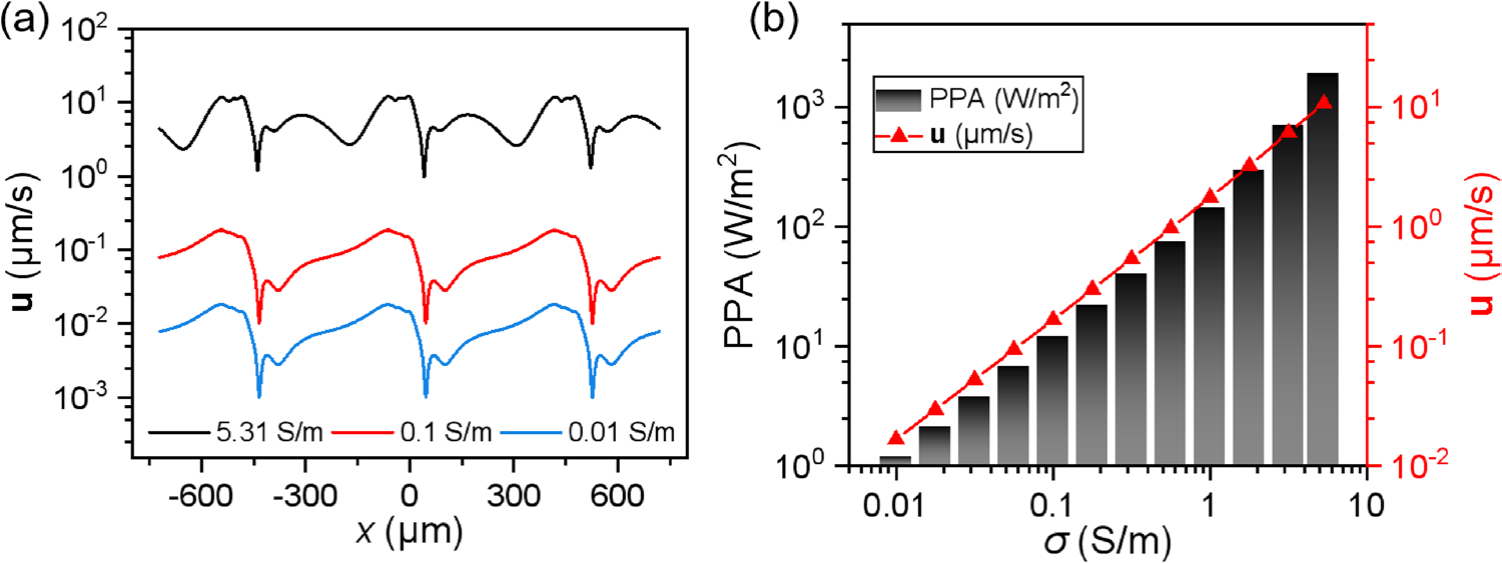
(**a**) Simulated fluid flow profile under different medium conductivities at a constant height of *y* = 20 µm. (**b**) The variation of PPA and fluid flow at one of experimental observation point at particle height of 20 µm as functions of the medium conductivity (*w*_1_ = 40 µm, *w*_2_ = 200 µm, *U*_eff_ = 1.3 V, *f* = 100 kHz).

As presented in Eqs. (1), (2) and (7), the fluid flow is primarily influenced by medium conductivity, voltage, and electrode geometry. The impact of the medium conductivity, as one of key parameters, on the fluid rate is studied at different observed positions. From Fig. 5a, the fluid fluctuates along electrode arrays (*x* direction) due to the difference of electric field strength on small and large electrodes, as illustrated in Fig. 3a. For the three selected medium conductivities, the velocity profiles show a similar shape at similar heights, *y* = 20 µm, implying a possible relation of the medium conductivity on the fluid flow (Fig. 5a). The fluid rate is almost linearly proportional to the medium conductivity (Fig. 5b). Moreover, the variation of power per unit area (PPA), which is defined as the current density multiplied by the electric potential, as a function of the medium conductivity was calculated to evaluate the energy efficiency of the antifouling system, given as:

13 $$ {\text{PPA}} = {\text{J}} \cdot \varphi . $$

Similar to the fluid flow, PPA also linearly depends on medium conductivity. This allows to predict the performance and energy consumption of the antifouling system for different work environments. Specifically, the antifouling system works with relatively high energy efficiency under low conductivity conditions (e.g., urban freshwater environments), but requires long time to move pollutants away from the system due to low flow velocities. Under high conductivity conditions (e.g., marine environments), high flow rate facilitates fast removal of surface sediments, whereas the energy consumption is correspondingly high. This further indicates that the proposed antifouling system possesses strong universal applicability under multiple working scenarios. To approach the computational results to the actual working conditions of the sensor (marine environment or high salinity lake water), subsequent calculations will be conducted under the conditions of 5.31 S/m. However, it is necessary to increase the electric frequency used in the calculation to 100 kHz to diminish the impact of unwanted ACEO (the calculated maximum ACEO flow rate under 5.31 S/m is achieved at 334 Hz but reduced to less than 1% of its peak value at *f* < 17 Hz or *f* > 6550 Hz)^15^.

**Figure 6:**
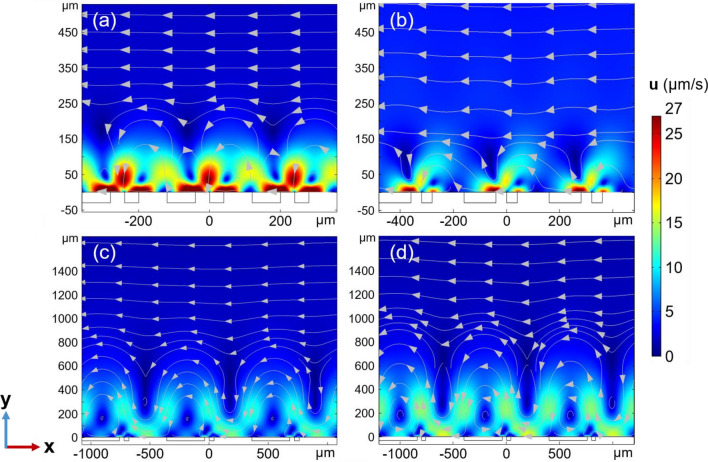
Numerical simulation of fluid flow profile at different aspect ratio: (**a**) *r* = 2, (**b**) *r* = 3, (**c**) *r* = 8, (**d**) *r* = 9. Note that the thickness of electrodes is constant for all aspect ratios (*w*_1_ = 40 µm, *U*_eff_ = 1.3 V, $\sigma_{{\text{m}}}$ = 5.31 S/m, and *f* = 100 kHz).

As mentioned above, the aspect ratio (*r*) describes the degree of asymmetry of the electrodes of the antifouling system. The asymmetry of the electrode array impacts the electric field intensity and temperature gradient and hence influences fluid flows induced by both ACET and buoyancy. Hence, we simulate the variation of the two-dimensional flow pattern as a function of* r*. As shown in Fig. 6 and Fig. S9, the asymmetric vortices with high flow velocities in the vicinity of the electrode surface prevail at small *r* (Fig. 6a,b) but decrease in strength with increasing *r* (Fig. S9). Instead, the symmetric vortices with low flow velocities above the electrode prevail at large *r* and further intensify with the enhancement of *r* (Fig. 6c,d). We believe that the existence of different flow vortex patterns with the variation of *r* is probably due to the change in the predominate role between ACET and buoyancy effects. The trajectory and residual rate of sediments were also calculated across various flow regimes, as depicted in Figs. S10 and S11. Compared to designs with a larger *r* (i.e., *r* = 8 or 9), sediments can be rapidly removed due to the pronounced asymmetry in the vortices when *r* is smaller (i.e., *r* = 2 or 3). However, a minuscule *r* introduces ‘stagnation zones’ and lower fluid velocity in the upper regions, resulting in a lower removal efficiency for *r* = 2 as compared to *r* = 3. We believe that the underlying mechanism is the competitive interaction between ACET and buoyancy.

**Figure 7:**
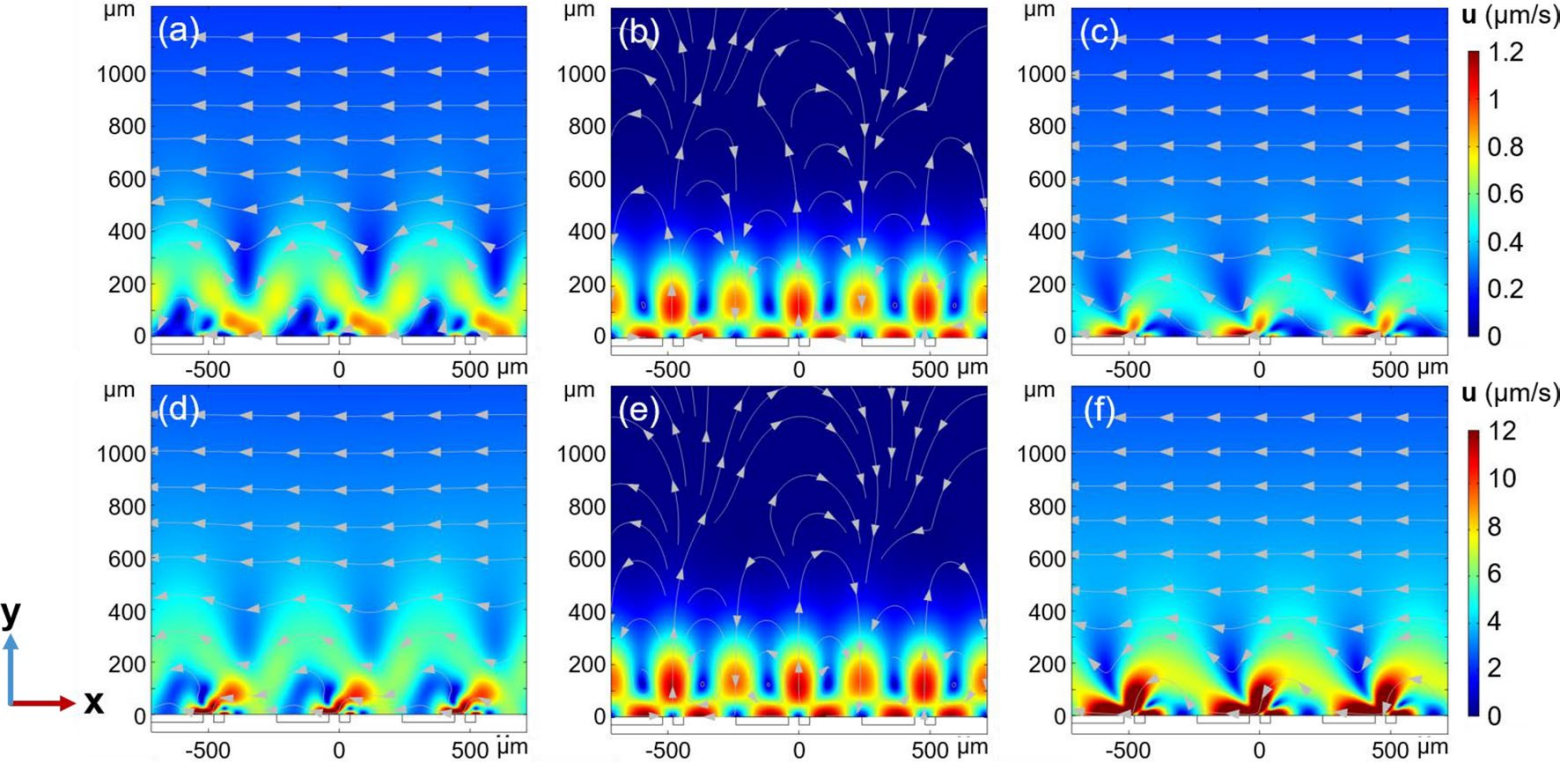
Simulated flow pattern at (**a–c**) 0.7 V and (**d–f**) 1.3 V input voltages. (**a,d**) The combined model including both ACET and buoyancy; (**b,e**) only the effect of buoyancy is considered and (**c,f**) only the effect of ACET is considered in the numerical model. (*w*_1_ = 40 µm, *r* = 5, $\sigma_{{\text{m}}}$ = 5.31 S/m, and *f* = 100 kHz).

To confirm the aforementioned hypothesis, we further simulate the impact of ACET and buoyancy on the flow distribution individually. The fluid flow exhibits symmetrical vortex states with a completely developed pattern (i.e., no unidirectional flow) when only buoyancy is simulated (Fig. 7b,e), and the system does not exhibit the capability to remove sediments (Figs. S12b, S13). Differently, the vortices created by ACET exhibit asymmetric flow undulations in the vicinity of the electrodes, and unidirectional net flow at positions away from the electrodes (Fig. 7c,f). This flow pattern caused by ACET, in combination with nDEP for most underwater sediments, can enable high-efficient removal of pollutants from underwater surfaces (Figs. S12c, S13). As previously mentioned, vortices dominated entirely by ACET exhibit ‘stagnation zones’, trapping a portion of the sediments, with the residual rate only being reduced to 7%. Further, buoyancy-induced fluid flow pattern do not change significantly with increasing input voltage from 0.7 (Fig. 7b) to 1.3 V (Fig. 7e). Concurrently, when only ACET is simulated, obvious enhancement of high flow velocity region in the vicinity of the electrodes were observed at high input voltage (Fig. 7f) compared to low voltage (Fig. 7c). As a result, increasing influence of ACET on the velocity in the combined model gives rise to the strengthened fluid disturbance at positions closed the electrode surfaces (Fig. 7a,d). Under the combined influence of ACET and buoyancy, the system achieved optimal particle removal efficiency, reducing the particle residual rate to zero after 400 s (Figs. S12a, S13). Further, it was found that the ACET is more sensitive to the variation of voltages than buoyancy. Such the sensitivity was then confirmed by the fitting curves presented in Fig. S14, in which the fluid rate induced by ACET is proportional to the voltage by power of 4.31, larger than the power of 2.93 of the voltage on the fluid flow induced by buoyancy.

**Figure 8:**
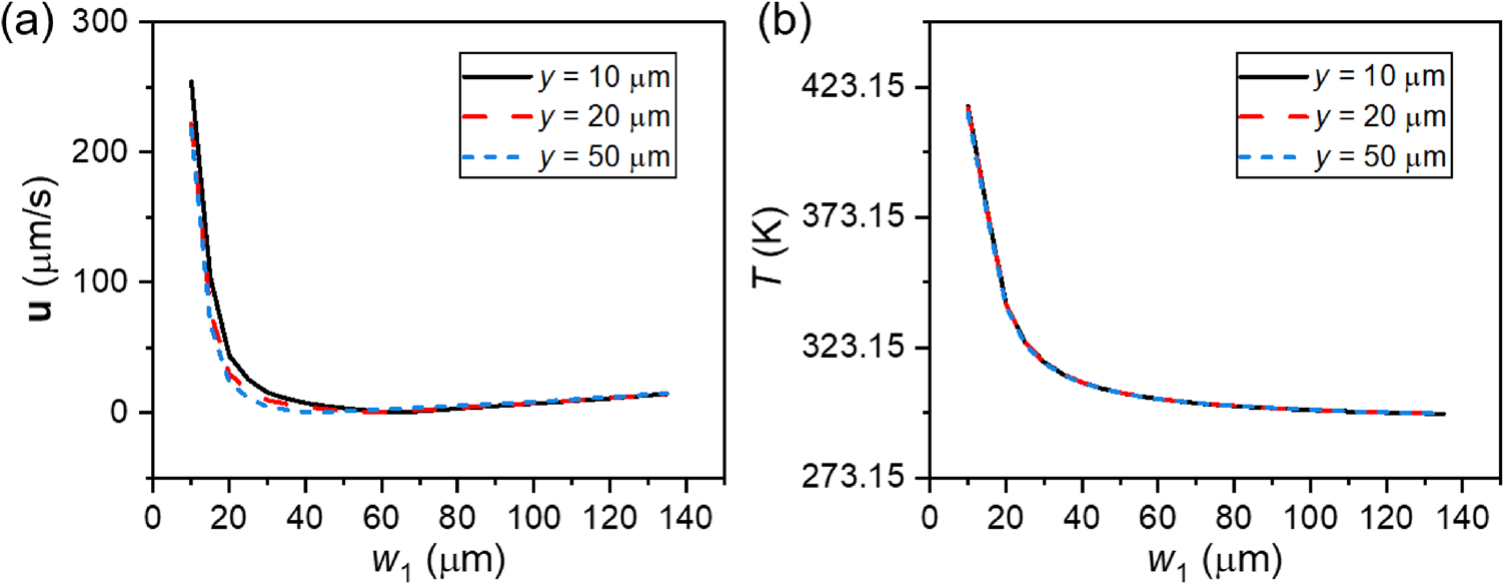
The numerical impact of short electrode width *w*_1_ on (**a**) velocity, (**b**) temperature at different height to the electrodes (*y* = 10, 20, 50 µm) (*r* = 5, *U*_eff_ = 1.1 V, $\sigma_{{\text{m}}}$ = 5.31 S/m, and *f* = 100 kHz).

The competitive relationship between ACET and buoyancy can also be clearly demonstrated at different electrode widths. As shown in Fig. 8a, the fluid velocity decreases sharply with increasing short-electrode width *w*_1_ for low *w*_1_, which is attributed to the rapid decrease in the electric field gradient. However, the fluid velocity increases thereafter with increasing *w*_1_ width, which can be ascribed to the rising buoyancy effect at large characteristic dimensions as mentioned in “Theory” section. The temperature variation at large *w*_1_ values as shown in Fig. 8b responds to the enhancement of buoyancy effect with enlarging the characteristic dimension of the system.

**Figure 9:**
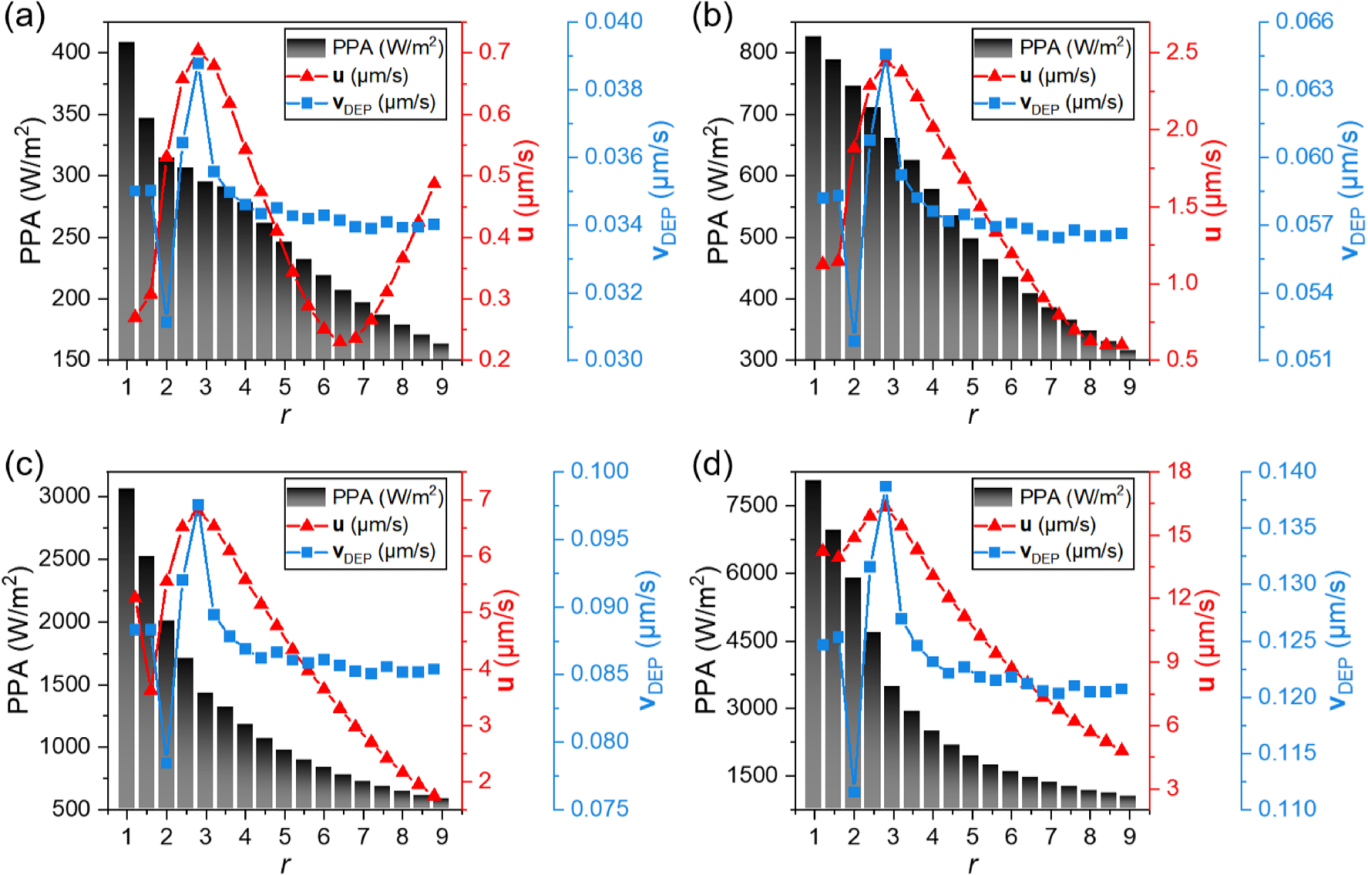
The impact of aspect ratio (*r*) on the calculated power per unit area (PPA), the fluid flow rate (**u**) and the DEP particle velocity (**v**_DEP_) respectively, at one of experimental observation point at particle height of 20 µm with different input voltages: (**a**) *U*_eff_ = 0.7 V; (**b**) *U*_eff_ = 0.9 V; (**c**) *U*_eff_ = 1.1 V; (**d**) *U*_eff_ = 1.3 V. (*w*_1_ = 40 µm, $\sigma_{{\text{m}}}$ = 5.31 S/m, and *f* = 100 kHz).

Since the antifouling system relies not only on the induced fluid flow, but also on the nDEP effect to levitate the sedimented particles away from optical surfaces, both fluid and DEP velocities are therefore studied over different aspect ratios. Clearly, there exists an optimal aspect ratio within the range of 2 to 3 under four different input voltages, at which both the fluid and DEP velocities reach maxima. The ACET flow decreases rapidly when *r* increases beyond the optimal value (Fig. 9a–d). Interestingly, the flow rate rebounds with aspect ratio at large *r* (e.g. *r* > 7), as observed from Fig. 9a and Fig. S15, depending on the input voltage. This could be attributed to the gradual dominance of the buoyancy effect against ACET with increasing characteristic dimensions of the system at low input voltages (e.g., *U*_eff_ = 0.7 V)^19^. Note that this inflection point clearly shifts towards large *r* with increasing *U*_eff_ (Fig. S15). Therefore, it can be inferred that the increase in voltage strengthens the effect of the vortices driven by ACET more than the vortices driven by buoyancy, as discussed above (Fig. S14). The aspect ratio greatly influences the DEP particle velocity at *r* < 3. Its impact on the DEP particle decreases with *r* values above 4. This combined behavior is due to the relatively strong variations of electric field gradient at small electrode spacing. Interestingly, the variation trend of **v**_DEP_ with *r* remains the same at different voltages (Fig. 9).

As an important reference indicator in electrokinetic systems, PPA was calculated to evaluate the relationship between antifouling performance and energy consumption of the proposed system (Fig. 9). As expected, PPA decreases rapidly with *r* at small *r* then slows down gradually with further increase of *r*. The antifouling system exhibits high energy consumption with poor performance at small aspect ratios (*r* < 3). Therefore, it is necessary to adopt asymmetric electrode design rather than symmetric electrode array to promote the energy efficiency of the proposed system.

At an aspect ratio of around 3, the fluid on the surface of electrodes is primarily driven by ACET, at which the buoyancy effect is low (see Fig. 6b). This configuration shows a strong unidirectional flow with both high ACET and DEP particle velocity, while at the same time the energy consumption (PPA) is considerably lower than for a symmetrical array (*r* = 1). When *r* is large enough to cross the inflection point of flow rate (Fig. 9a, Fig. S15), the fluid flow on the surface of the antifouling system will be driven by buoyancy, at which the fluid rate continues to increase with increasing the aspect ratio. In this case, the system works with sufficiently low energy consumption, whereas the weakened unidirectional flow rate and nDEP force attenuates its capability for removing sedimented pollutants. On the other hand, the fluid rate and DEP particle velocity remains at their maximum regardless of voltage at the aspect ratio of around 3. In this case, high performance removal of surface sediments at low energy consumption is possible through regulating the operating voltage of the antifouling system.

## Conclusions

In summary, we proposed a novel underwater surface antifouling strategy based on ACEK. The antifouling system is composed of groups of asymmetric electrodes to initiate negative DEP force and unidirectional ACET flow to resuspend and remove particles deposited in the system. ITO was chosen as electrode material to maintain the transparency of optical surfaces. A theoretical model that strongly couples electrostatic, fluid and temperature fields was established to predict the Joule heating induced ACET and buoyancy flows as well as trajectories of submicron PS particles within the system. Numerical simulations confirmed the necessity of the asymmetric electrode design, where the ACET induces obvious asymmetric vortex flow in the vicinity of electrode surfaces and strong unidirectional flow away from the electrodes. Experimental measurement of trajectories of 0.75 µm PS fluorescent microspheres demonstrated the theoretical predictions with good accuracy. The performance of the as-designed antifouling system was numerically evaluated thereafter through variations of electrode structure and system operating parameters. As expected, the aspect ratio, which characterises the degree of electrodes asymmetry, greatly influences the flow pattern. It was found that ACET dominates the overall fluid flow at small aspect ratios, at which an optimal aspect ratio of around 3 can be identified with desired flow profile and energy efficiency. At large aspect ratios buoyancy-driven flow dominates over ACET, at which the system works with comparatively low energy consumption but poor antifouling performance due to significantly attenuated unidirectional flow and nDEP force on pollutants removal. Overall, the universal strategy proposed in this work is expected to provide theoretical basis for tailoring design of advanced optical surface antifouling systems with high antifouling performance and low energy consumption. The efficacy of electrokinetic cells in mitigating biological fouling was evaluated by subjecting two identical cells (Fig. S4) to a 25-day aquarium test to validate the underlying principle. After this duration, it was evident that the slide with the electric field (Fig. S16a) exhibited significantly less biological coverage compared to the one without the field (Fig. S16b), thereby confirming the effectiveness of the electrokinetic effect in suppressing biological fouling.

### Supplementary Information

Supplementary Figures.

## Data Availability

The datasets generated an analysed during the current study are available from the corresponding author on reasonable request.
