# Supplementary material for: A universal AC electrokinetics-based strategy toward surface antifouling of underwater optics
Source: Sci Rep. 2024 Jul 12;14:16125. doi: 10.1038/s41598-024-66251-2 (PMC11245552; doi:10.1038/s41598-024-66251-2)
Supplement: Supplementary file 1 — Supplementary Figures. [file 41598_2024_66251_MOESM1_ESM.docx]

**Appendix A. Supplementary Information:**

**A universal AC electrokinetics-based strategy toward surface antifouling of underwater optics**

Hao Jiang^1^, Yan Wang^1,^*, Fei Du^2^, Stefan Stolte^2^, Uwe Specht^3^, Georg R. Pesch^4^, Michael Baune^5,^*

^1^ College of Chemistry and Chemical Engineering, Qingdao University, 266071 Qingdao, P. R. China

^2^ Institute of Water Chemistry, Dresden University of Technology, 01069 Dresden, Germany

^3^ The Fraunhofer Institute for Manufacturing Technology and Advanced Materials, 28359 Bremen, German

^4^ School of Chemical and Bioprocess Engineering, University College Dublin, Belfield, Dublin 4, Ireland

^5^ Center for Environmental Research and Sustainable Technology, University of Bremen, 28359 Bremen, Germany

* Corresponding authors

*E**-mail address:* [yanwang@qdu.edu.cn](mailto:yanwang@qdu.edu.cn) (Dr. Yan Wang); [mbaune@uni-bremen.de](mailto:mbaune@uni-bremen.de) (Dr. Michael Baune)


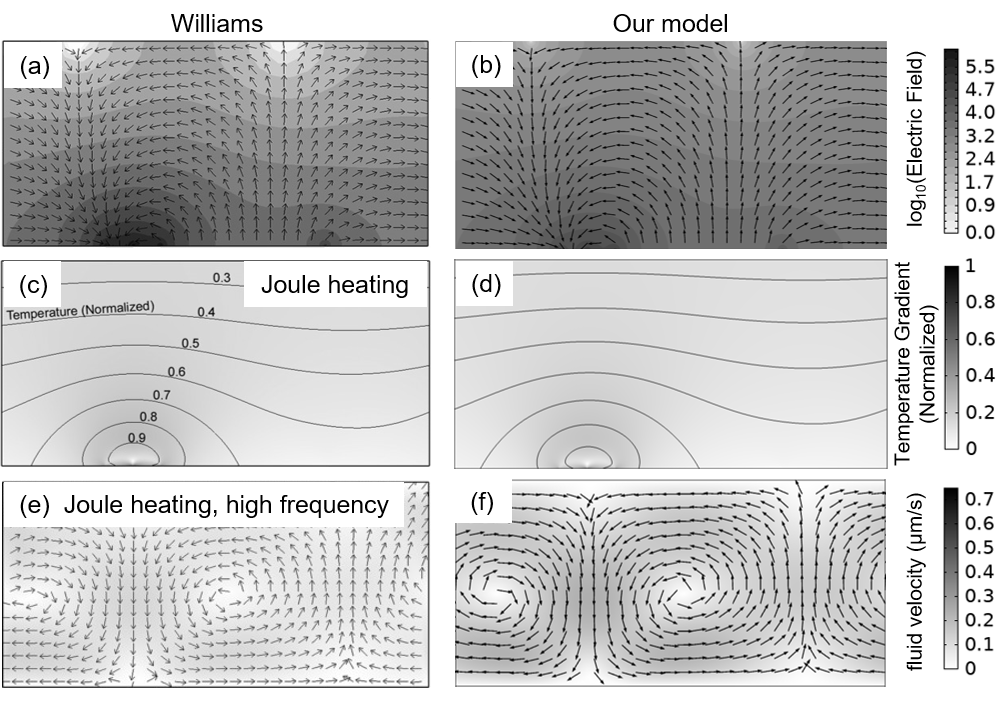


**Figure S1.** Comparison of simulation results with literature: (a, c, e) calculations from Williams and (b, d, f) calculations using the proposed model from this work; (a, b) electric field; (c, d) temperature field; (e, f) fluid flow field. (*U*_eff_ = 1 V, $\sigma$ = 1 S/m, and *f* = 2*10^10^ Hz for the above simulations.)


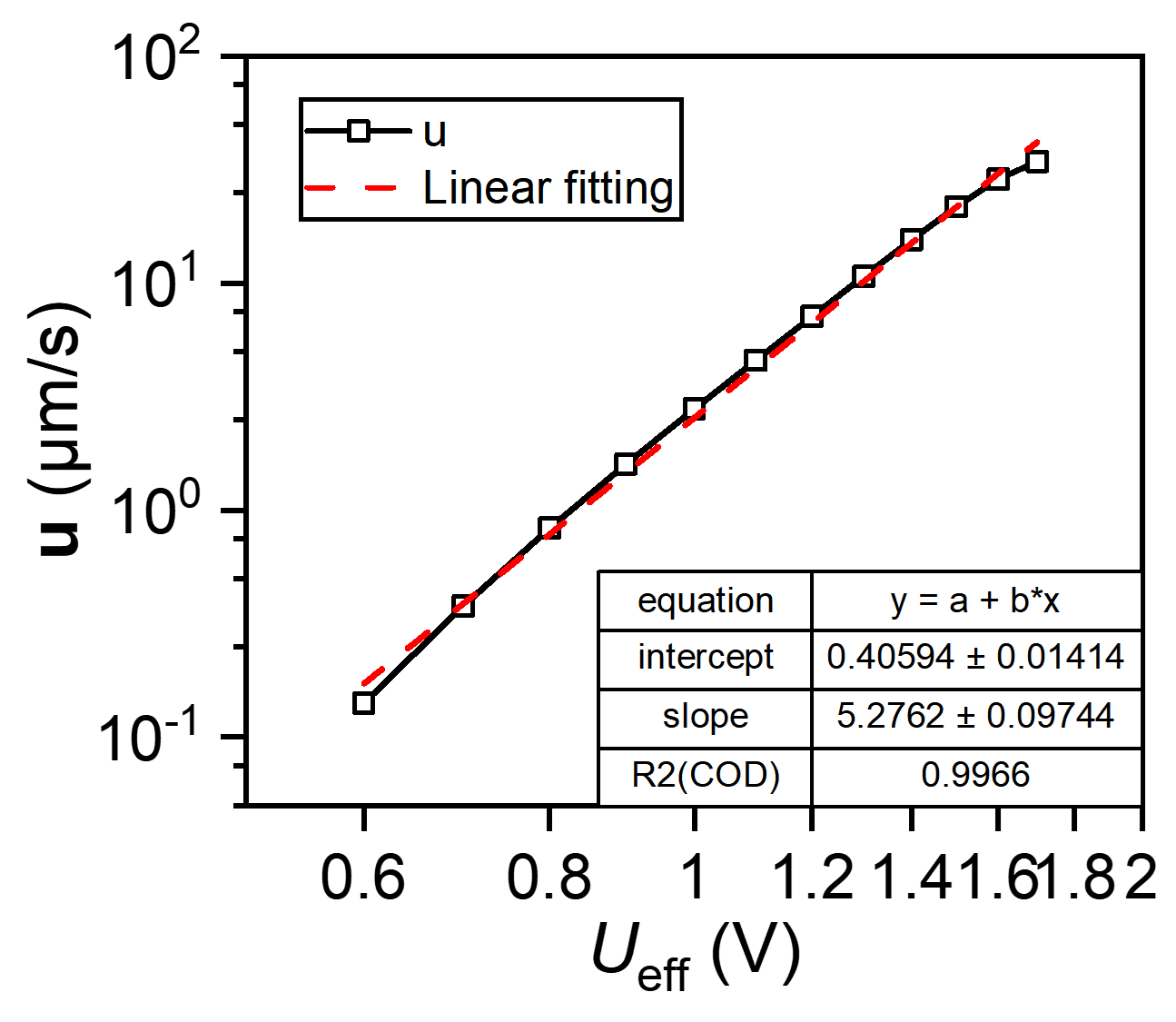


**Figure S2.** Linear fitting relationship between velocity and applied voltage. (*w*_1_ = 40 µm, *r* = 5, *σ* = 5.31 S/m, and *f* = 100 kHz)


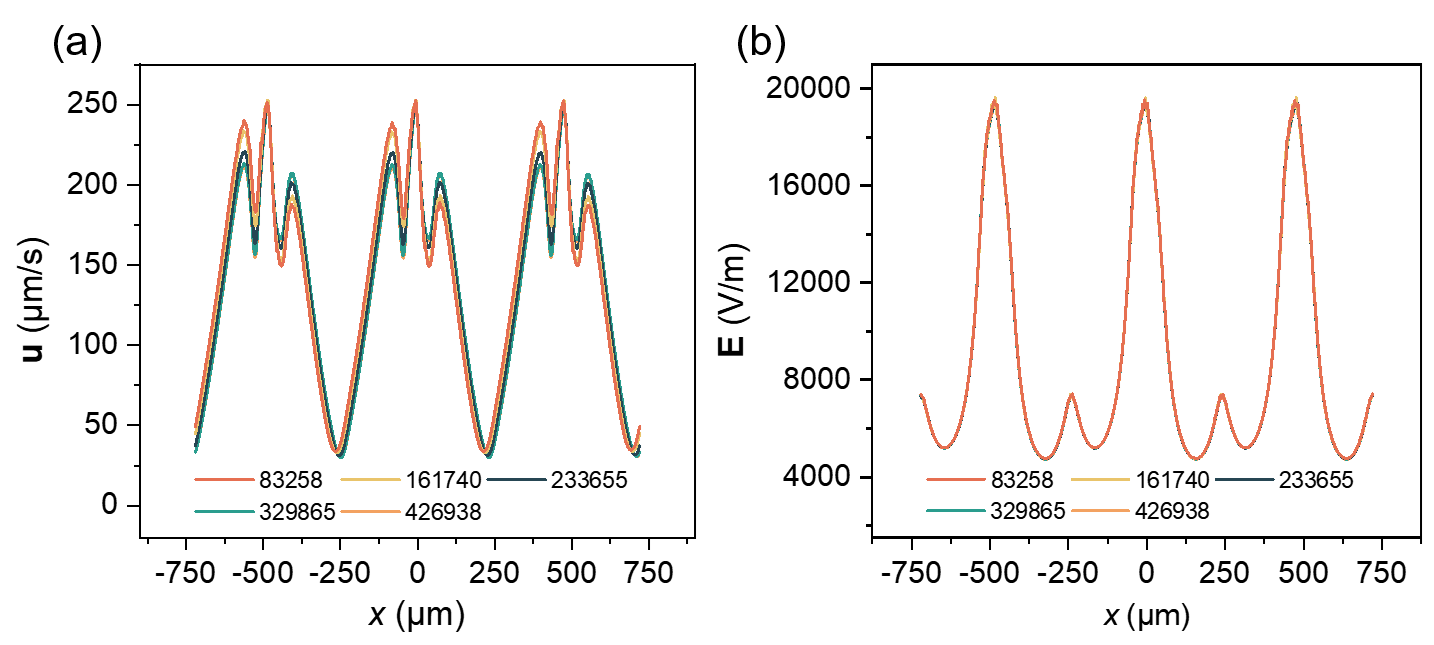


**Figure S3.** Mesh independence validation: distribution of (a) fluid flow rate, (b) electric field. (*w*_1_ = 40 µm, *y* = 25 µm, *w*_1_: *w*_2_ = 5, *U*_eff_ = 2 V, $\sigma$ = 5.31 S/m, and *f* = 100 kHz)


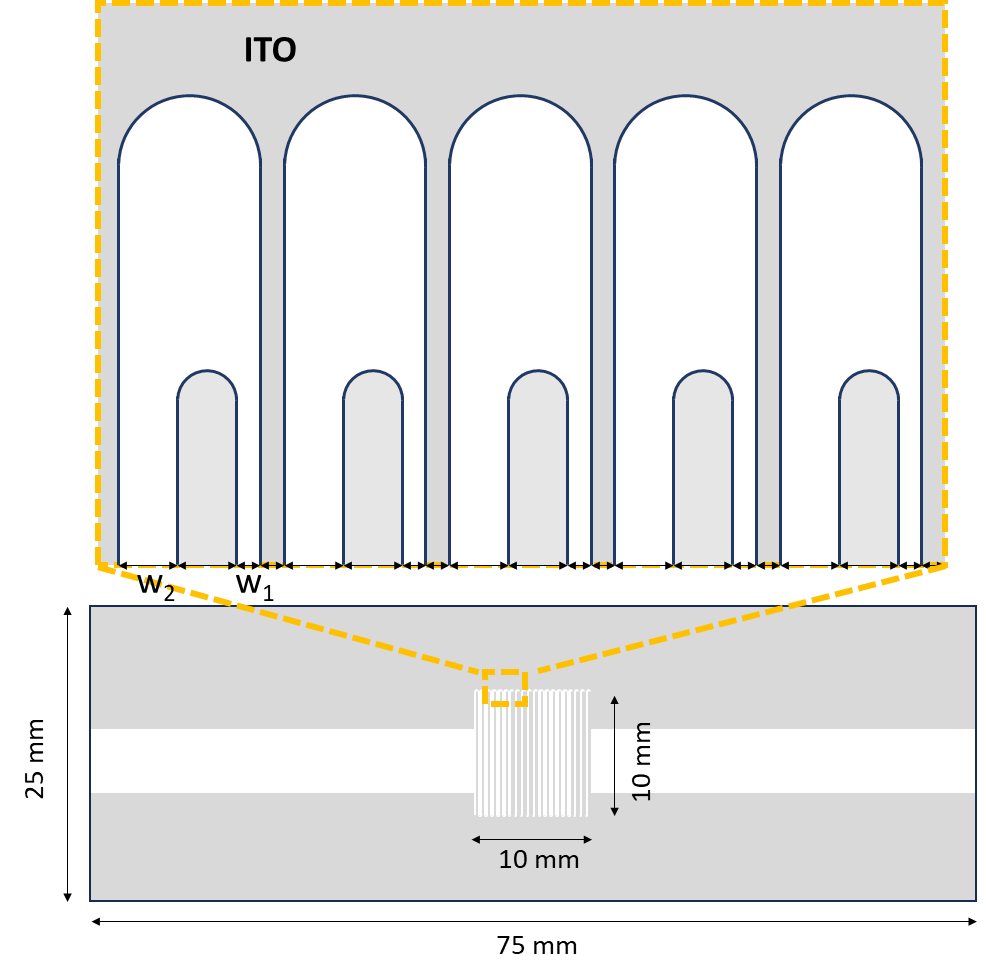


**Figure S4.** A schematic representation of an electrokinetic cell on an ITO-coated glass slide (75 x 25 mm) with selectively laser ablated ITO (10 x 10 mm) to create interdigitated electrodes array with electrode ratio of 5 (w_1_= 40 µm, w_2_ = 200 µm).


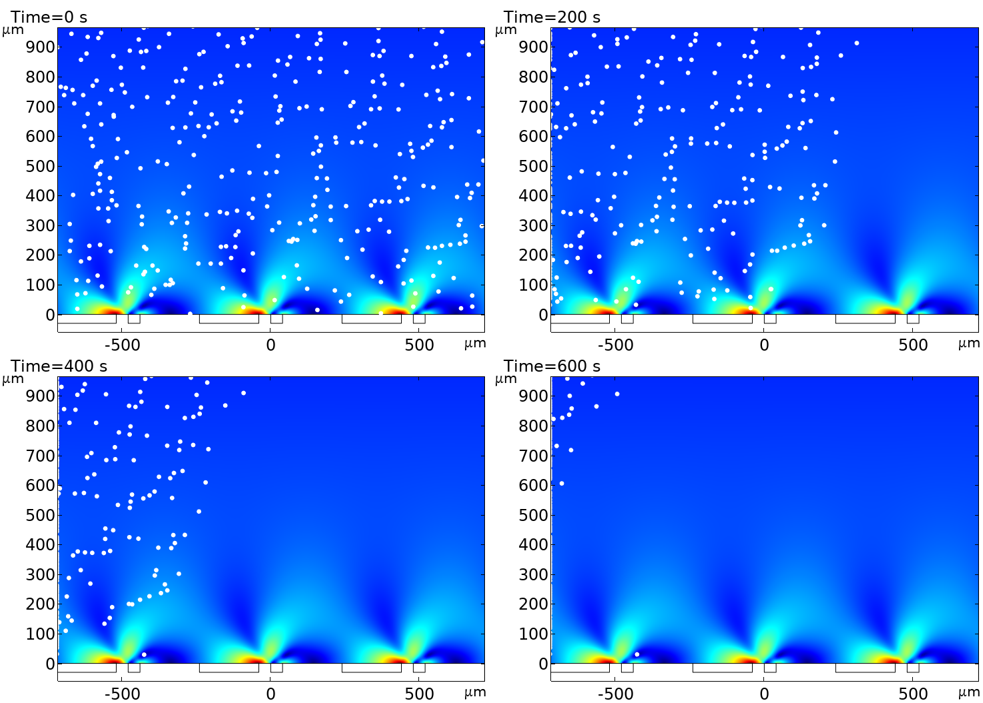


**Figure S5.** Snapshots of particle positions at different time. (*w*_1_ = 40 µm, *w*_2_ = 200 µm, *U*_eff_ = 5.66 V, $\text{σ}_{\text{m}}$ = 0.01 S/m, *f* = 1 kHz, and *a* = 0.75 µm)

**Figure S6.** The calculated Re[$\text{K}\left( \text{ω} \right)$] of PS particles as a function of electric frequency for different medium conductivity.


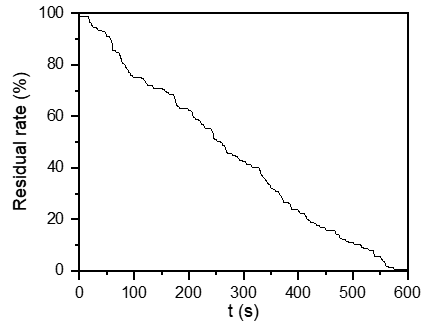


**Figure S7.** The variation in particle residual rate (defined as the particle count at a given time/that at t = 0 s) within the 0.5 mm area on device surface. (*w*_1_ = 40 µm, *w*_2_ = 200 µm, *U*_eff_ = 5.66 V, $\text{σ}_{\text{m}}$ = 0.01 S/m, *f* = 1 kHz, and *a* = 0.75 µm)


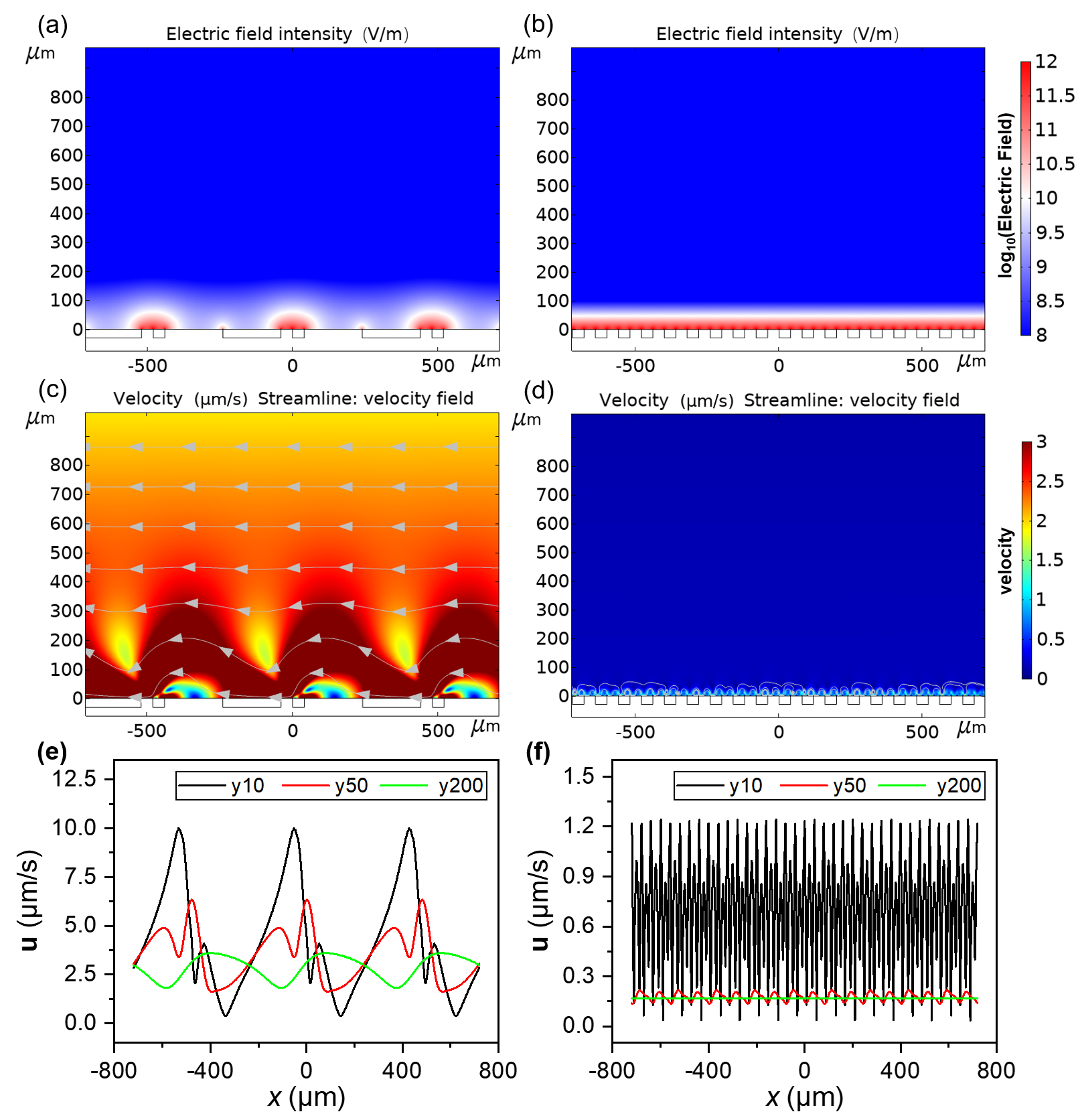


**Figure S8.** Numerical simulations of distributions of (a) electric field, *r* = 5; (b) electric field, *r* = 1; (c) flow rate, *r* = 5 and (d) flow rate, *r* = 1. (*w*_1_ = 40 µm, *U*_eff_ = 5.66 V, $\sigma$ = 0.01 S/m, and *f* = 1 kHz)


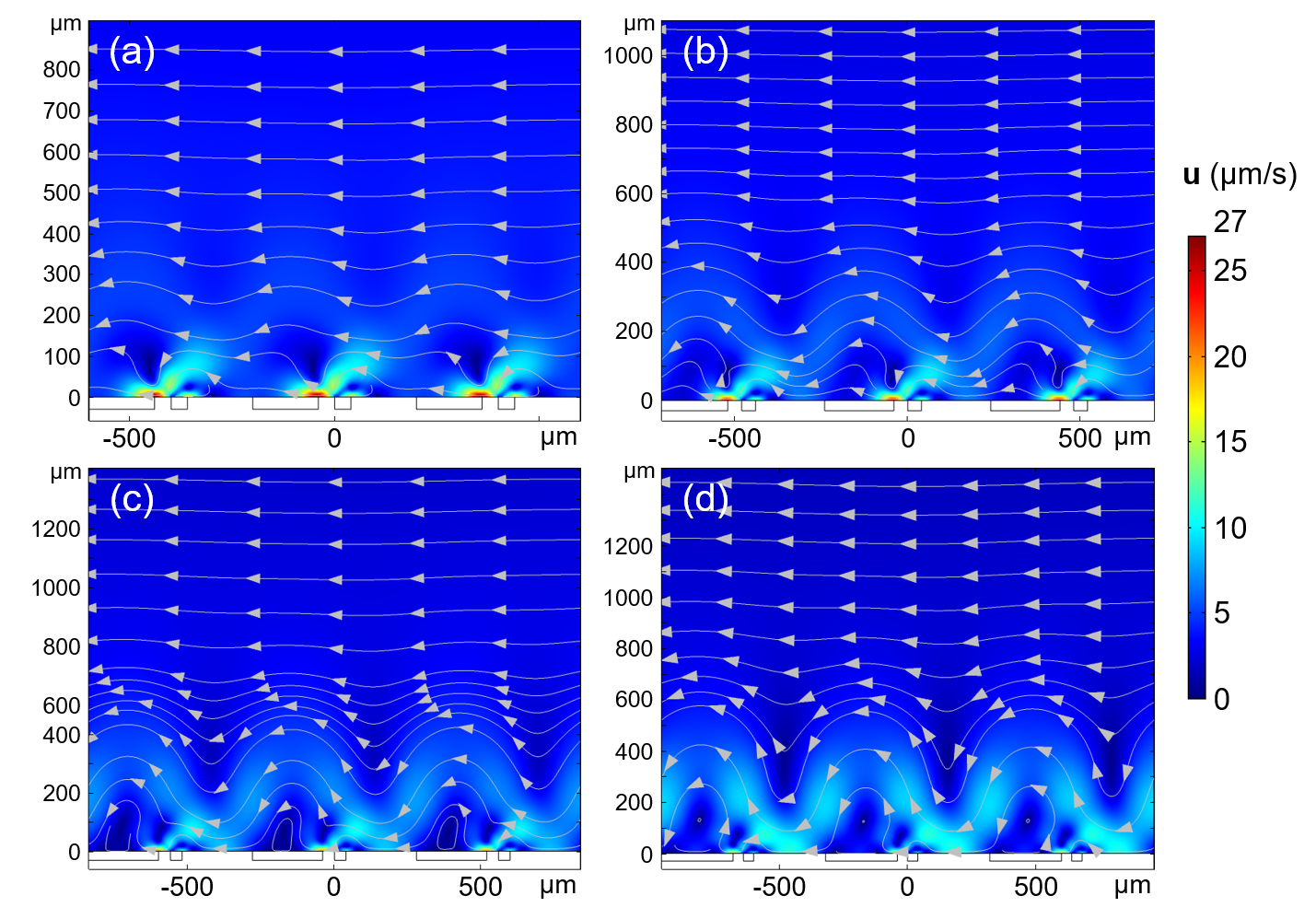


**Figure S9.** Numerical simulation of fluid flow profile at different aspect ratio: (a) to (d) represents *r* = 4~7, respectively. (*w*_1_ = 40 µm, *U*_eff_ = 1.3 V, $\sigma$ = 5.31 S/m, and *f* = 100 kHz)


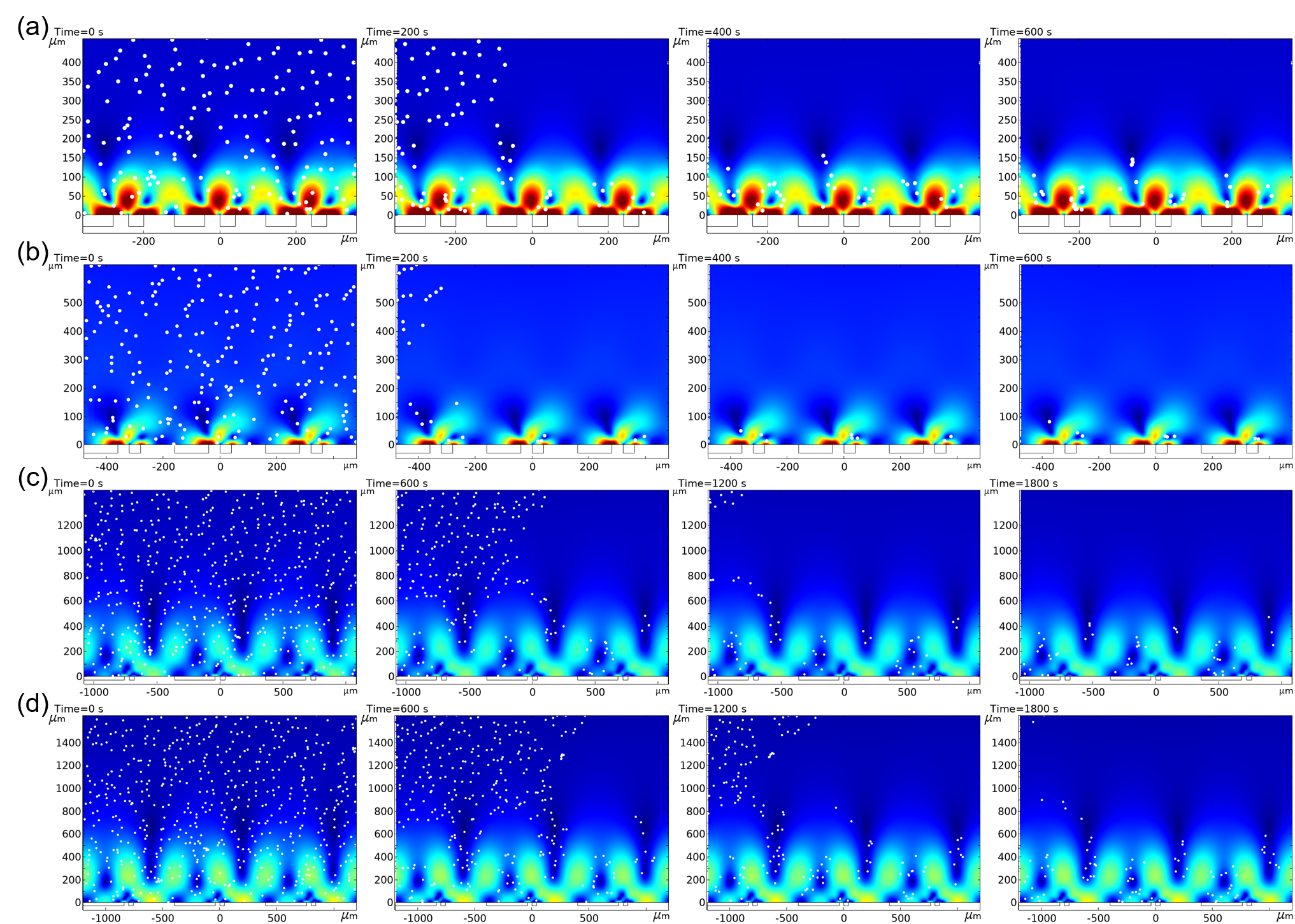


**Figure S10.** Snapshots of particle positions at different time and aspect ratio: (a) *r* = 2, (b) *r* = 3, (c) *r* = 8, (d) *r* = 9. (*w*_1_ = 40 µm, *U*_eff_ = 1.3 V, $\text{σ}_{\text{m}}$ = 5.31 S/m, *f* = 100 kHz, and *a* = 0.75 µm)


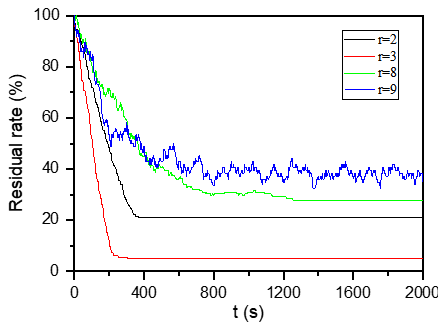


**Figure S11.** The variation in particle residual rate within the 0.5 mm area on device surface under different aspect ratio. (*w*_1_ = 40 µm, *U*_eff_ = 1.3 V, $\text{σ}_{\text{m}}$ = 5.31 S/m, *f* = 100 kHz, and *a* = 0.75 µm)


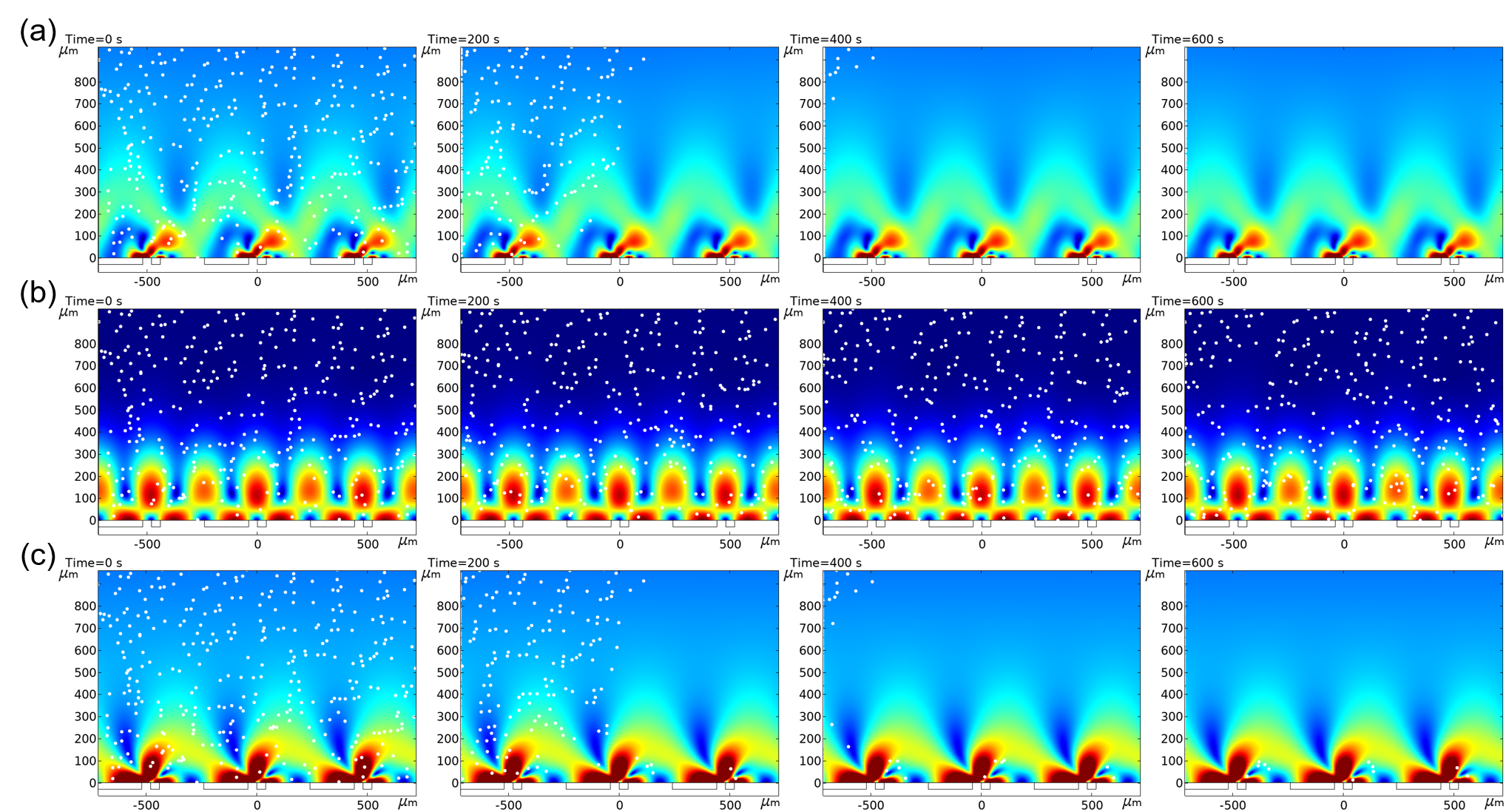


**Figure S12.** Snapshots of particle positions at different time when considering (a) the composite model, (b) buoyancy, and (c) ACET. (*w*_1_ = 40 µm, *r* = 5, *U*_eff_ = 1.3 V, $\text{σ}_{\text{m}}$ = 5.31 S/m, *f* = 100 kHz, and *a* = 0.75 µm)


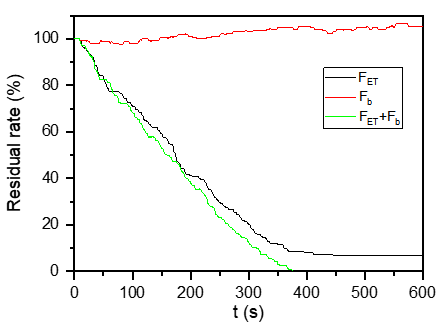


**Figure S13.** The variation in particle residual rate within the 0.5 mm area on device surface when considering the competitive interactions between buoyancy and ACET. (*w*_1_ = 40 µm, *r* = 5, *U*_eff_ = 1.3 V, $\text{σ}_{\text{m}}$ = 5.31 S/m, *f* = 100 kHz, and *a* = 0.75 µm)

**Figure S14.** Linear fitting relationship between velocity and applied voltage when (a) only ACET is applied and (b) only buoyancy is applied. (*w*_1_ = 40 µm, *r* = 5, $\sigma$ = 5.31 S/m, and *f* = 100 kHz)


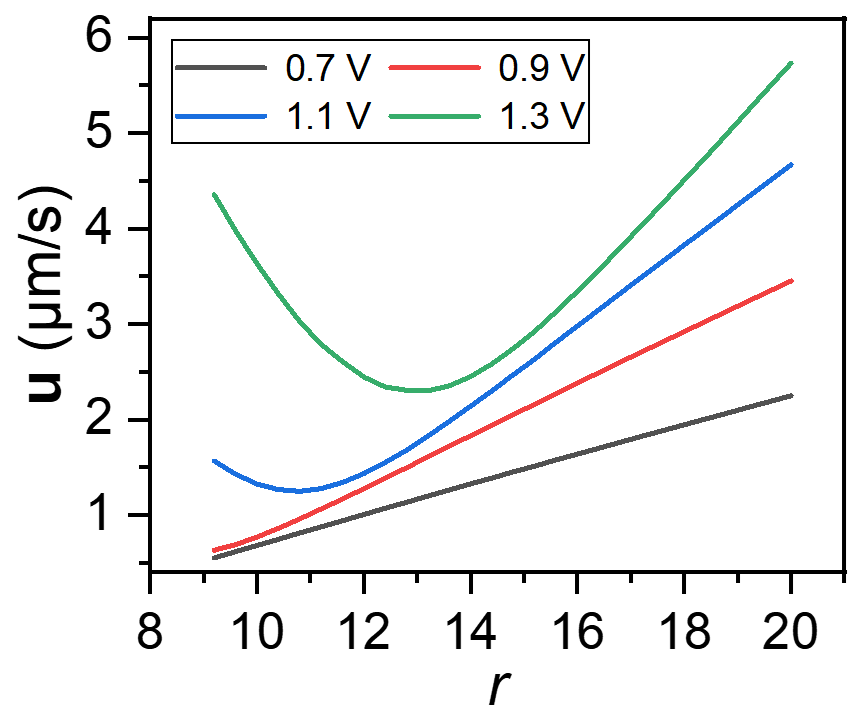


**Figure S15.** The impact of aspect ratio (*r*) on the fluid flow rate (**u**) at one of experimental observation point at particle height of 20 µm with different input voltages when *r* > 9. (*w*_1_ = 40 µm, $\sigma$ = 5.31 S/m, and *f* = 100 kHz)


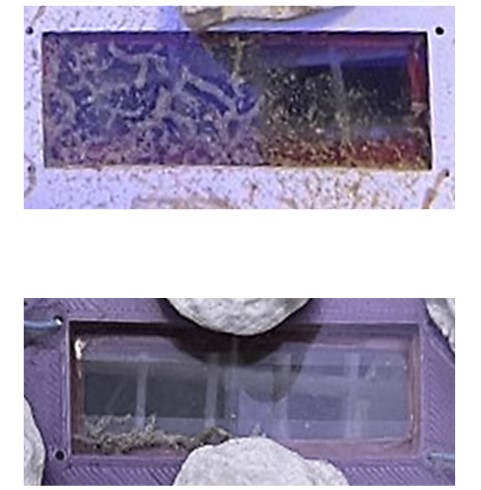


a)

b)

**Figure S16.** Comparison of biological fouling on identical electronic cells (shown in Figure S4) with electric field (2 V_eff_, 200 kHz) (a) and without electric field (b) in an aquarium with seawater for 25 days.
